# Endothelial *Cdk5* deficit leads to the development of spontaneous epilepsy through CXCL1/CXCR2-mediated reactive astrogliosis

**DOI:** 10.1084/jem.20180992

**Published:** 2019-11-07

**Authors:** Xiu-xiu Liu, Lin Yang, Ling-xiao Shao, Yang He, Gang Wu, Yu-huan Bao, Nan-nan Lu, Dong-mei Gong, Ya-ping Lu, Tian-tian Cui, Ning-he Sun, Dan-yang Chen, Wei-xing Shi, Kohji Fukunaga, Hong-shan Chen, Zhong Chen, Feng Han, Ying-mei Lu

**Affiliations:** 1 Key Laboratory of Cardiovascular & Cerebrovascular Medicine, School of Pharmacy, Nanjing Medical University, Nanjing, China; 2 Institute of Pharmacology and Toxicology, College of Pharmaceutical Sciences, Zhejiang University, Hangzhou, China; 3 Department of Physiology, School of Basic Medical Sciences, Nanjing Medical University, Nanjing, China; 4 School of Medicine, Zhejiang University City College, Hangzhou, Zhejiang, China; 5 Departments of Pharmaceutical, Administrative, and Basic Sciences, Schools of Pharmacy and Medicine, Loma Linda University Health, Loma Linda, CA; 6 Department of Pharmacology, Graduate School of Pharmaceutical Sciences, Tohoku University, Sendai, Japan; 7 Center for Global Health of Nanjing Medical University, Nanjing, China

## Abstract

Blood–brain barrier (BBB) dysfunction has been suggested to play an important role in epilepsy. However, the mechanism mediating the transition from cerebrovascular damage to epilepsy remains unknown. Here, we report that endothelial cyclin-dependent kinase 5 (CDK5) is a central regulator of neuronal excitability. Endothelial-specific *Cdk5* knockout led to spontaneous seizures in mice. Knockout mice showed increased endothelial chemokine (C-X-C motif) ligand 1 (Cxcl1) expression, decreased astrocytic glutamate reuptake through the glutamate transporter 1 (GLT1), and increased glutamate synaptic function. Ceftriaxone restored astrocytic GLT1 function and inhibited seizures in endothelial *Cdk5*-deficient mice, and these effects were also reversed after silencing Cxcl1 in endothelial cells and its receptor chemokine (C-X-C motif) receptor 2 (Cxcr2) in astrocytes, respectively, in the CA1 by AAV transfection. These results reveal a previously unknown link between cerebrovascular factors and epileptogenesis and provide a rationale for targeting endothelial signaling as a potential treatment for epilepsy.

## Abstract (precis)

Liu et al. reveal a key mechanism that mediating the transition from cerebrovascular damage to epilepsy. They identify the endothelial cyclin-dependent kinase 5 (CDK5) regulates astrocytic glutamate reuptake and increased glutamate synaptic function through CXCL1/CXCR2-mediated astrogliosis.

## Graphical Abstract

**Figure:**
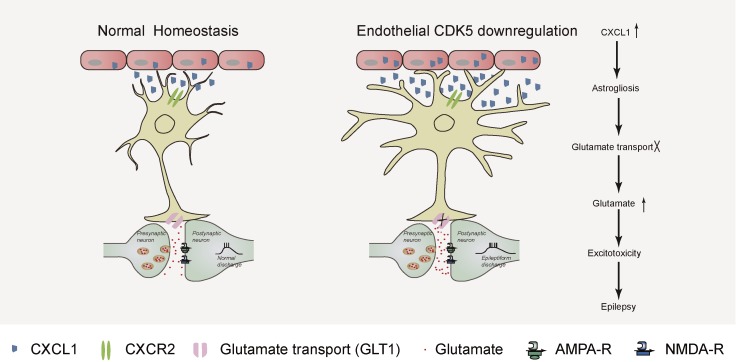

## Introduction

Epilepsy, characterized by recurrent seizures, affects 65–70 million people worldwide (Moshé et al., 2015; Thijs et al., 2019). Although the hyperexcitability underlying epilepsy is believed to be caused by an imbalance of synaptic excitation and inhibition (Li et al., 2011; Neumann et al., 2017; Paz and Huguenard, 2015), antiepileptic strategies directly targeting neuronal excitability have proven to be insufficient in a significant proportion of patients (Löscher and Schmidt, 2006; Eyo et al., 2017; Ferlazzo et al., 2017). This insufficiency points to the need to identify the cause of the imbalance between excitation and inhibition. Drugs targeting the underlying mechanism of this imbalance may prove to be more effective than current antiepileptic medications.

The microvasculature at the blood–brain barrier (BBB) plays an important role in the maintenance of brain homeostasis (Obermeier et al., 2013; Tran et al., 2016). BBB microvascular dysfunction has been suggested to contribute to brain disorders including epilepsy (Blanchette and Daneman, 2015; Obermeier et al., 2013). However, the molecular events linking microvascular pathology to epilepsy remain elusive (Han et al., 2017). Cyclin-dependent kinase 5 (CDK5) is important in several biological processes, including cell proliferation (Pu et al., 2017), sprouting (Tian et al., 2010), and migration (Lampropoulou et al., 2018; Liebl et al., 2010). CDK5 inhibition suppresses angiogenesis in hepatocellular carcinoma (Herzog et al., 2016) and human endothelial cells (ECs; Rárová et al., 2018), and retards the development of endothelial senescence and atherosclerosis (Bai et al., 2012). Endothelial-specific *Cdk5* KO in mice also inhibits melanoma tumor growth and improves the sensitivity to anti-angiogenic treatment (Merk et al., 2016).

Until now, research focusing on microvascular function in epilepsy has not been extensive, and experimental microvascular pathology–related epilepsy models are lacking. In this study, we found that endothelial-specific *Cdk5* KO in mice induced spontaneous hippocampal epileptic discharges in an age-dependent manner. Our evidence further suggests that endothelial *Cdk5* deletion down-regulates astrocytic GLT1-mediated current through endothelial chemokine (C-X-C motif) ligand 1 (CXCL1) and its receptor chemokine receptor 2 (CXCR2)-induced progressive reactive astrogliosis. The reduced GLT1 function increases glutamate synaptic current and, thus, may contribute to the development of epilepsy.

## Results and discussion

### Endothelial conditional deletion of *Cdk5* induces spontaneous seizures

**Figure S1.**
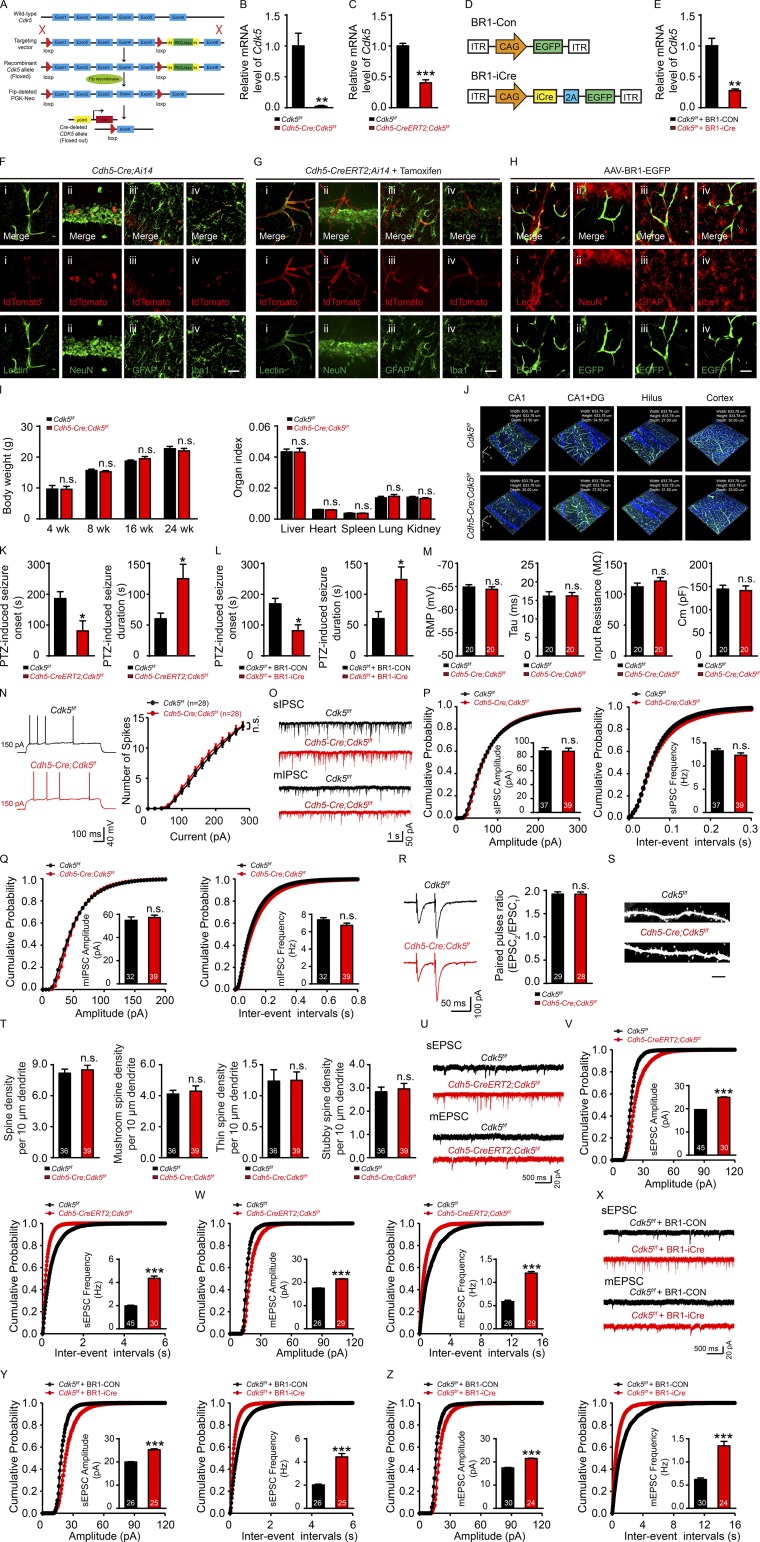
**Ablation of endothelial *Cdk5* increased sensitivity to PTZ-induced epilepsy. (A)** Schematic diagram of the strategy used to create conditional endothelial-specific *Cdk5* KO (*Cdh5-Cre;Cdk5^f/f^*) mice. **(B)** The *Cdk5* mRNA level in primary cultured ECs from the cerebral microvessels of *Cdh5-Cre;Cdk5^f/f^* and *Cdk5^f/f^* mice was normalized to the corresponding Gapdh level (*n* = 4 mice per group; **, P < 0.01; unpaired two-tailed Student’s *t* test). **(C)** Relative mRNA level in primary cultured ECs from the cerebral microvessels of *Cdk5* in *Cdh5-CreERT2;Cdk5^f/f^* and *Cdk5^f/f^* mice (*n* = 3 mice per group; ***, P < 0.001; unpaired two-tailed Student’s *t* test). **(D)** Schematic diagram of AAV-BR1 constructs indicating the inverted terminal repeats (ITR) at both ends, a CAG promoter-driven EGFP (BR1-Con), or the CAG promoter-driven iCre linked by the 2A peptide to EGFP (BR1-iCre). **(E)** The relative mRNA level of *Cdk5* in primary cultured ECs from the cerebral microvessels was evaluated in two groups (*Cdk5^f/f^* + BR1-Con group and *Cdk5^f/f^* + BR1-iCre group; *n* = 3 mice per group; **, P < 0.01; unpaired two-tailed Student’s *t* test). **(F and G)** Z-stack images of Rosa26-tdTomato expression (Cre reporter) in the hippocampus of 4-wk-old *Cdh5-Cre;Ai14* reporter mice (F) and 5-wk-old *Cdh5-CreERT2;Ai14* reporter mice (mice were injected with tamoxifen for 3 consecutive d starting at 4 wk; G), costained with Lectin (i), NeuN (ii), GFAP (iii), and Iba1 (iv). Representative images from three independent experiments are shown. Bars, 20 µm. **(H)** Representative Z-stack images of EGFP expression in the hippocampus of *Cdk5^f/f^* mice with BR1-Con injection, costained with Lectin (i), NeuN (ii), GFAP (iii), and Iba1 (iv). Representative images from three independent experiments are shown 3 wk after vector injection. BR1-Con–transduced cells (green) were positive for the endothelial marker Lectin (red). Bar, 20 µm. **(I)** The effect of conditional *Cdk5* KO on body weight and the organ index (*n* = 5 mice per group, unpaired two-tailed Student’s *t* test). **(J)** Z-stack images of vascular morphology between *Cdh5-Cre;Cdk5^f/f^* and control mice. **(K)** Quantitative results of seizure onset and duration triggered by PTZ in 5-wk-old *Cdh5-CreERT2;Cdk5^f/f^* and *Cdk5^f/f^* mice (*n* = 5 mice each group; *, P < 0.05; unpaired two-tailed Student’s *t* test). **(L)** Quantitation of seizure onset and duration induced by PTZ in two groups at 7 wk (*Cdk5^f/f^* + BR1-Con group and *Cdk5^f/f^* + BR1-iCre group; *n* = 5 mice per group, *, P < 0.05; unpaired two-tailed Student’s *t* test). **(M)** Quantitative results of intrinsic membrane property recordings in the hippocampus of *Cdh5-Cre;Cdk5^f/f^* and control mice at 4 wk (*n* = 5 mice per group; unpaired two-tailed Student’s *t* test). **(N)** Representative APs (left) and number of spikes (right) in the medial prefrontal cortices of *Cdh5-Cre;Cdk5^f/f^* and control mice at 4 wk (*n* = 5 mice per group; two-way ANOVA followed by Tukey’s multiple comparisons test). **(O)** Traces showing sIPSC and mIPSC recorded in CA1 of hippocampus slices from *Cdh5-Cre;Cdk5^f/f^* and control mice. **(P and Q)** Cumulative distribution plots of the amplitudes and inter-event intervals of sIPSC (P) and mIPSC (Q). The insets show the average sIPSC and mIPSC amplitudes and frequencies (*n* = 5 mice per group; unpaired two-tailed Student’s *t* test). **(R)** Representative traces of PPR recordings performed in the hippocampus and quantification of PPR in *Cdh5-Cre;Cdk5^f/f^* and control mice at 4 wk (*n* = 5 mice per group, unpaired two-tailed Student’s *t* test). **(S)** Representative spine density in apical dendrites of hippocampal pyramidal neurons in *Cdh5-Cre;Cdk5^f/f^* and control mice at 4 wk. Bar, 5 µm. **(T)** Quantification of total and mature spine densities (*n* = 5 mice of each group, unpaired two-tailed Student’s *t* test). **(U)** Traces showing sEPSC and mEPSC recorded in the hippocampus of *Cdh5-CreERT2;Cdk5^f/f^* and *Cdk5^f/f^* mice. **(V and W)** Cumulative distribution plots of the amplitudes and inter-event intervals of sEPSC (V) and mEPSC (W) from 5-wk-old *Cdh5-CreERT2;Cdk5^f/f^* and *Cdk5^f/f^* mice. The insets show the average amplitudes and frequencies of sEPSC and mEPSC in two groups (*n* = 3–5 mice per group; ***, P < 0.001; unpaired two-tailed Student’s *t* test). **(X)** Traces showing sEPSC and mEPSC recorded in the hippocampus of 7-wk-old BR1-Con– or BR1-iCre–injected *Cdk5^f/f^* mice. **(Y and Z)** Cumulative distribution plots of the amplitudes and inter-event intervals of sEPSC (Y) and mEPSC (Z) from BR1-Con– or BR1-iCre–injected *Cdk5^f/f^* mice. The insets show the average amplitudes and frequencies of sEPSC and mEPSC in two groups (*n* = 3–5 mice per group; ***, P < 0.001; unpaired two-tailed Student’s *t* test). The numbers inside the bars represent the numbers of cells from three to five mice. Error bars represent means ± SEM; n.s., not significant.

To investigate the role of endothelial *Cdk5* in brain, we generated three sets of conditional endothelial-specific *Cdk5* KO mice (Tan et al., 2019; *Cdh5-Cre;Cdk5^f/f^* mice, *Cdh5-CreERT2;Cdk5^f/f^* mice, BR1-iCre-*Cdk5^f/f^* mice; Fig. S1, A–E). First, to determine the specificity of *Cre* recombinase expression in ECs, the *Cdh5-Cre* or *Cdh5-CreERT2* mice line was crossed with the *Ai14* reporter mice line (Rosa-CAG-LSL-tdTomato-WPRE::deltaNeo). We found that tdTomato was expressed almost exclusively in Lectin^+^ (an EC marker) ECs (Fig. S1, F and G), indicating sufficient and specific Cre expression in ECs. Second, brain microvasculature EC (BMVEC)–targeted adeno-associated virus (AAV; termed AAV-BR1) was further used to delete target molecules solely in brain ECs (Körbelin et al., 2016). Consistent with previous reports, robust enhanced GFP (EGFP) expression was largely restricted to the brain vasculature in AAV-BR1-EGFP–injected mice (Fig. S1 H). Moreover, the KO had no effect on body weight, the organ index (Fig. S1 I), or brain vascular morphology (Fig. S1 J).

**Figure 1.**
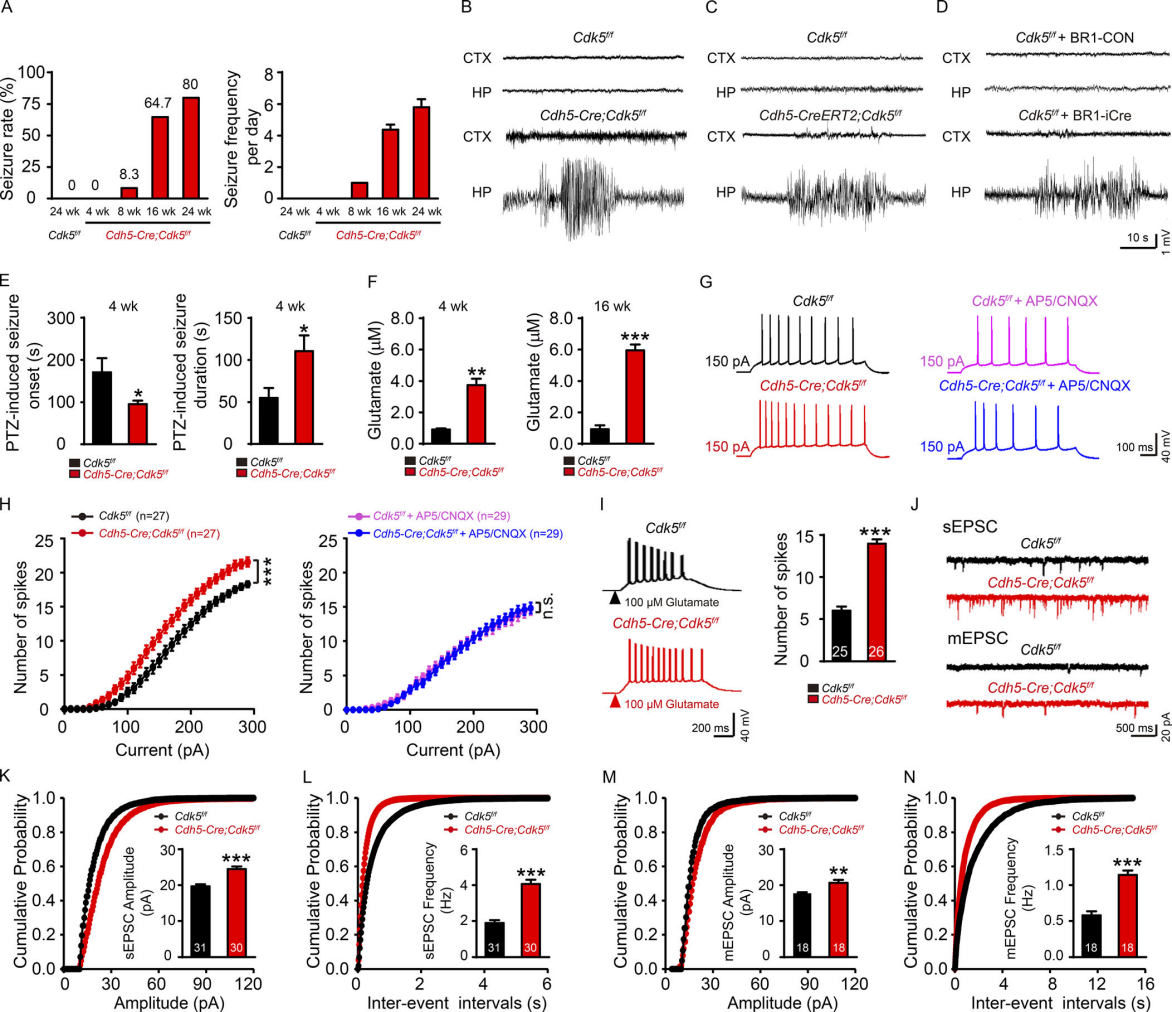
**Conditional deletion of endothelial *Cdk5* induces spontaneous seizures. (A)** Spontaneous seizure rates and frequencies in *Cdh5-Cre;Cdk5^f/f^* and *Cdk5^f/f^* mice at different ages. **(B–D)** Typical EEGs recorded from the cortex (CTX) and hippocampus (HP) in *Cdk5^f/f^* and *Cdh5-Cre;Cdk5^f/f^* mice (B) or *Cdh5-CreERT2;Cdk5^f/f^* mice (C) and *Cdk5^f/f^* mice injected with BR1-Con or BR1-iCre (D). **(E)** PTZ-induced seizure onset latency and duration in *Cdh5-Cre;Cdk5^f/f^* and *Cdk5^f/f^* mice at 4 wk (*n* = 5 mice per group; *, P < 0.05; unpaired two-tailed Student’s *t* test). **(F)** Hippocampal glutamate concentrations in *Cdh5-Cre;Cdk5^f/f^* and *Cdk5^f/f^* mice at 4 wk (left) and 16 wk (right; *n* = 4–5 mice per group; **, P < 0. 01; ***, P < 0.001; unpaired two-tailed Student’s *t* test). **(G)** Example traces of AP responses to positive current injection treatment at 150 pA with or without AP5 (20 mM) and CNQX (40 mM) in 4-wk-old *Cdh5-Cre;Cdk5^f/f^* and *Cdk5^f/f^* mice. **(H)** Quantification across 0–300-pA current injections in 10-pA steps (*n* = 5 mice per group; ***, P < 0.001; two-way ANOVA followed by Tukey’s multiple comparisons test). **(I)** Representative traces and AP firing frequencies induced by 500-ms treatment with 100 µM glutamate in *Cdh5-Cre;Cdk5^f/f^* and *Cdk5^f/f^* mice (*n* = 5 mice per group; ***, P < 0.001; unpaired two-tailed Student’s *t* test). **(J)** Traces showing sEPSC and mEPSC recorded in *Cdh5-Cre;Cdk5^f/f^* and *Cdk5^f/f^* mice. **(K–N)** Cumulative probability plots summarizing the mean sEPSC amplitudes (K) and sEPSC inter-event intervals (L) and cumulative probability plots summarizing the mean mEPSC amplitudes (M) and mEPSC inter-event intervals (N) in *Cdh5-Cre;Cdk5^f/f^* and *Cdk5^f/f^* mice at 4 wk. The insets depict the average sEPSC and mEPSC amplitudes and frequencies (*n* = 5 mice per group; **, P < 0.01; ***, P < 0.001; unpaired two-tailed Student’s *t* test). The numbers inside the bars represent the numbers of cells from five mice. The data are presented as means ± SEM. n.s., not significant.

C*dh5-Cre;Cdk5^f/f^* mice showed an age-dependent increase in the prevalence and frequency of seizures using 24-h video surveillance (Fig. 1 A). Epileptic seizures were also confirmed by local field potential recordings in the hippocampus in 11 of 17 16-wk-old *Cdh5-Cre;Cdk5^f/f^* mice (Fig. 1 B and Video 1). No significant electroencephalographic (EEG) changes were observed in the cortex (Fig. 1 B). Spontaneous seizures were not observed in control littermates. Furthermore, epileptic waves were also recorded in 6 of 10 8-wk-old *Cdh5-CreERT2;Cdk5^f/f^* mice (treated with tamoxifen at 4 wk; Fig. 1 C) and in 6 of 12 8-wk-old *Cdk5^f/f^* mice (i.v. injection with BR1-iCre virus at 4 wk; Fig. 1 D) in the hippocampus. No spontaneous seizures were observed in 4-wk-old *Cdh5-Cre;Cdk5^f/f^* mice. For mice with no spontaneous seizures, their sensitivity to the convulsant drug pentylenetetrazol (PTZ) was measured. Interestingly, our data showed a decrease in onset time (95.40 ± 8.20 s vs. 171.00 ± 32.83 s) and an increase in duration of seizures induced by PTZ (110.50 ± 18.74 s vs. 55.00 ± 11.75 s; Fig. 1 E) compared with control (*Cdk5^f/f^*) mice. Similar results were observed in *Cdh5-CreERT2;Cdk5^f/f^* mice (Fig. S1 K) and BR1-iCre–injected *Cdk5^f/f^* mice (Fig. S1 L). These results suggest that endothelial *Cdk5* deficit leads to the development of spontaneous epilepsy.

**Video 1.** **An episode of a spontaneous behavioral seizure was observed in 16-wk-old *Cdh5-Cre;Cdk5^f/f^* mice.** The mouse displays spontaneous seizures with salivation, masticatory jaw movements, and falling, which correspond with the EEG spiking (high frequency and high amplitude) Frame rate, 25.00 frames per second.

The increase in hippocampal discharges may have been caused by an imbalance between excitatory and inhibitory neurons (Lopez-Santiago et al., 2017). Using microdialysis in freely moving mice, we found a significant increase in extracellular glutamate in the hippocampus in *Cdh5-Cre;Cdk5^f/f^* mice (Fig. 1 F). Whole-cell recordings in brain slices showed an increased excitability of hippocampal CA1 pyramidal neurons in *Cdh5-Cre;Cdk5^f/f^* mice. There were no significant differences in intrinsic membrane properties of pyramidal neurons between the groups at 4 wk (Fig. S1 M). This increase in excitability was blocked by the glutamate receptor antagonist DL-2-amino-5-phosphonovaleric acid (DL-AP5) and 6-cyano-7-nitroquinoxaline-2,3-dione (Fig. 1, G and H). In contrast, no significant changes in excitability were observed in medial prefrontal cortex pyramidal neurons (Fig. S1 N). Hippocampal CA1 pyramidal neurons also showed an increased response to exogenously applied glutamate (100 µM; Fig. 1 I), and an increase in both the amplitudes and frequencies of spontaneous and miniature excitatory postsynaptic current (sEPSC and mEPSC, respectively; Fig. 1, J–N). Endothelial *Cdk5* KO, however, had no effect on spontaneous and miniature inhibitory postsynaptic current (sIPSC and mIPSC, respectively; Fig. S1, O–Q). The lack of changes in the paired pulse ratio (PPR) of evoked EPSC (Fig. S1 R) indicated that the increases in the mEPSC and sEPSC frequencies were not attributed to increases in presynaptic release probability. There were also no changes in the morphology or density of dendritic spines in CA1 pyramidal neurons between 4-wk-old mutant and control mice (Fig. S1, S and T). Similarly, the increased amplitudes and frequencies of sEPSC/mEPSC were also observed in *Cdh5-CreERT2;Cdk5^f/f^* mice (treated with tamoxifen at 4 wk; Fig. S1, U–W) and *Cdk5^f/f^* mice (i.v. injection with BR1-iCre virus at 4 wk; Fig. S1, X–Z).

These data suggested that selective deletion of *Cdk5* in ECs increases CA1 neuronal hyperexcitability and seizure generation. More importantly, the increases in both the amplitudes and frequencies of sEPSC and mEPSC suggested that synaptic levels of glutamate were higher in *Cdh5-Cre;Cdk5^f/f^* mice than in *Cdk5^f/f^* mice. However, no significant PPR changes or normal spine morphology were observed in *Cdh5-Cre;Cdk5^f/f^* mice. Therefore, we propose that abnormal extracellular excitatory neurotransmitter levels trigger hyperexcitability of pyramidal neurons of the hippocampus in endothelial *Cdk5*-deficient mice at 4 wk.

### Deletion of endothelial *Cdk5* induces progressive astrogliosis and impairs astroglial GLT1 function

**Figure 2.**
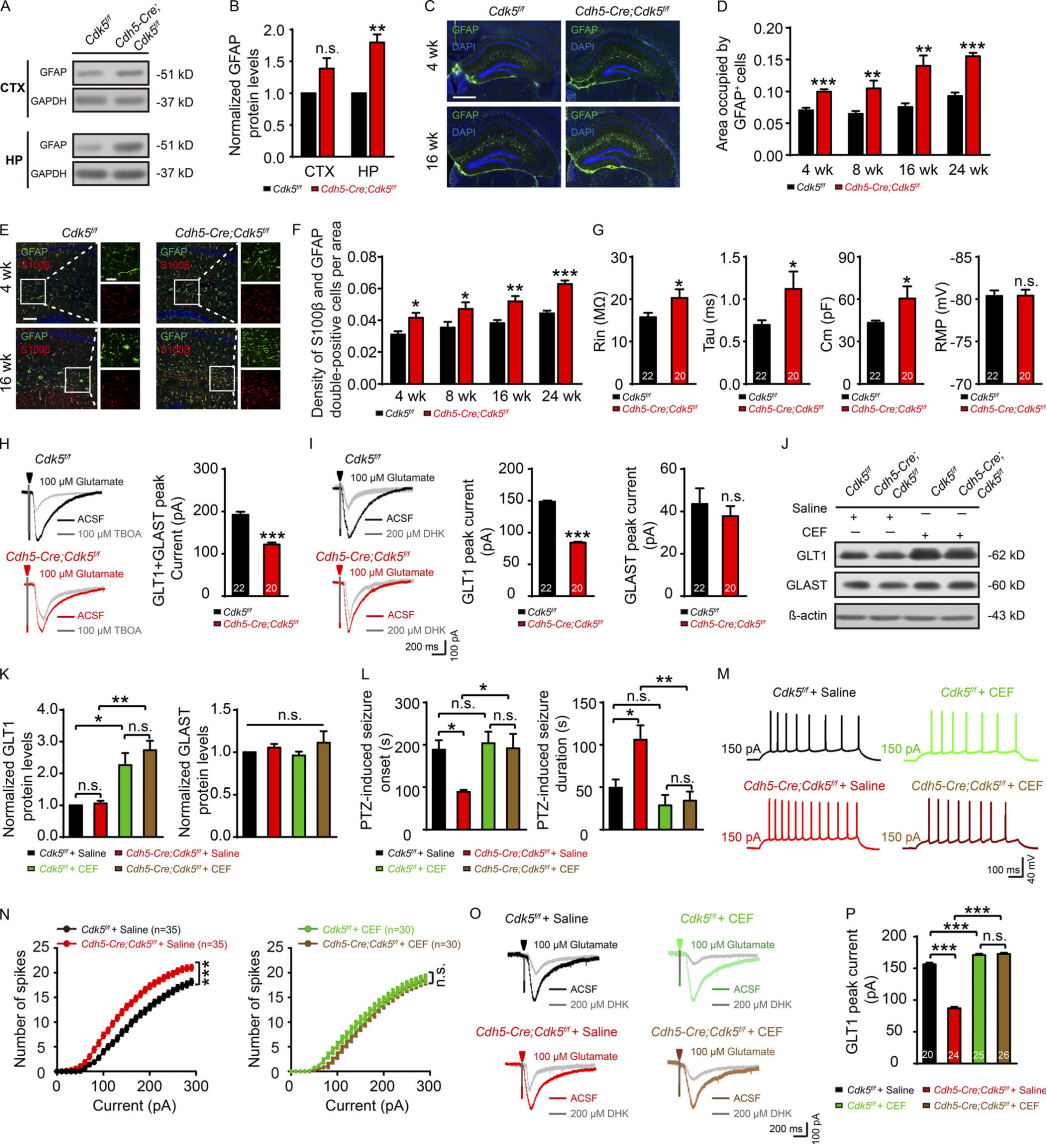
**Endothelial *Cdk5* deletion causes progressive astrogliosis and decreased astrocytic glutamate uptake. (A and B)** Representative Western blot of GFAP (A) and quantification of GFAP expression (B) in the cortex (CTX) and hippocampus (HP) in *Cdh5-Cre;Cdk5^f/f^* and *Cdk5^f/f^* mice at 16 wk (*n* = 3 mice per group; **, P < 0.01; unpaired two-tailed Student’s *t* test). **(C–F)** Representative stitched images of immunostaining for GFAP (green; C) and S100β (red; E) in the hippocampus in *Cdh5-Cre;Cdk5^f/f^* and *Cdk5^f/f^* mice at 4 and 16 wk. DAPI staining is shown in blue. Quantification of the area occupied by GFAP^+^ cells (D) and the density of S100β and GFAP double-positive cells (F) in *Cdh5-Cre;Cdk5^f/f^* and *Cdk5^f/f^* mice at the ages of 4, 8, 16, and 24 wk (*n* = 3 mice per group; *, P < 0.05; **, P < 0.01; ***, P < 0.001; unpaired two-tailed Student’s *t* test). Bars: C, 500 µm; E: main images, 100 µm; insets, 50 µm. **(G)** Intrinsic membrane properties, including R_in_, τ_m_ (Tau), C_m_, and RMP recorded in hippocampal astrocytes from brain slices in *Cdh5-Cre;Cdk5^f/f^* and *Cdk5^f/f^* mice at 4 wk (*n* = 5 mice per group; *, P < 0.05; unpaired two-tailed Student’s *t* test). **(H and I)** Glutamate uptake by astrocytes was assessed after puff application of 100 µM glutamate for 500 ms before and after the application of 100 µM TBOA (H) or 200 µM DHK (I) in brain slices in *Cdh5-Cre;Cdk5^f/f^*and *Cdk5^f/f^* mice at 4 wk. Quantification of transport currents associated with TBOA-sensitive glutamate uptake (H, right) and DHK-sensitive glutamate uptake (I, right) in brain slices in *Cdh5-Cre;Cdk5^f/f^* and control mice at 4 wk (*n* = 5 mice per group; ***, P < 0.001; unpaired two-tailed Student’s *t* test). **(J and K)** Representative Western blots of GLT1 and GLAST in astrocytes (J) and quantification of GLT1 and GLAST expression (K) in *Cdh5-Cre;Cdk5^f/f^* and *Cdk5^f/f^* mice treated with saline or CEF at 4 wk (*n* = 5 mice per group; *, P < 0.05; **, P < 0.01; one-way ANOVA followed by Tukey’s multiple comparisons test). **(L)** PTZ-induced seizure onset latency and duration in *Cdh5-Cre;Cdk5^f/f^* and *Cdk5^f/f^* mice treated with saline or CEF at 4 wk (*n* = 5 mice per group; *, P < 0.05; **, P < 0.01; one-way ANOVA followed by Tukey’s multiple comparisons test). **(M)** Representative traces of AP responses to positive current injection treatments at 150 pA with saline or CEF in *Cdh5-Cre;Cdk5^f/f^* and *Cdk5^f/f^* mice at 4 wk. **(N)** Quantification across 0–300-pA current injections in 10-pA steps (*n* = 5 mice per group; ***, P < 0.001; two-way ANOVA followed by Tukey’s multiple comparisons test). **(O)** Representative GLT1 transport currents following DHK incubation in the hippocampus from 4-wk-old *Cdh5-Cre;Cdk5^f/f^* and *Cdk5^f/f^* mice with or without CEF. **(P)** Quantification of the effect of CEF administration on GLT1 transport currents (*n* = 5 mice per group; ***, P < 0.001; one-way ANOVA followed by Tukey’s multiple comparisons test). The numbers inside the bars represent the numbers of cells from five mice. The bars with error bars represent means ± SEM; n.s., not significant.

**Figure S2.**
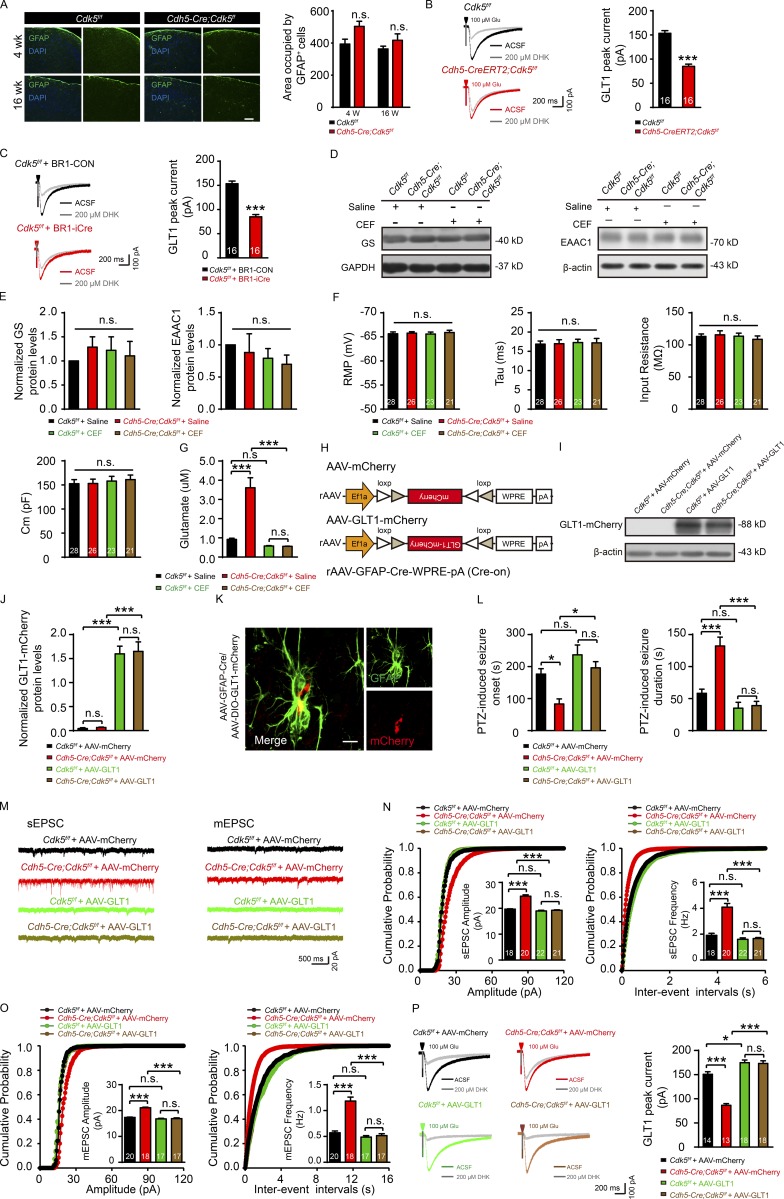
**Dysfunction of astroglial GLT1 contributes to neuronal excitability and epilepsy. (A)** Representative images of GFAP (green) and DAPI (blue) staining (left) and quantification of the area occupied by GFAP^+^ cells (right) in the cortices of *Cdh5-Cre;Cdk5^f/f^* and *Cdk5^f/f^* mice at 4 and 16 wk (*n* = 3 mice of each group; unpaired two-tailed Student’s *t* test). Bar, 100 µm. **(B and C)** Representative GLT1 transport current incubated with DHK (left) and quantification of GLT1 transport current (right) in the hippocampus in 5-wk-old *Cdh5-CreERT2;Cdk5^f/f^* or *Cdk5^f/f^* mice (B) and 7-wk-old BR1-Con– or BR1-iCre–injected *Cdk5^f/f^* mice (C; *n* = 3 mice per group; ***, P < 0.001; unpaired two-tailed Student’s *t* test). **(D)** Representative image showing the effect of CEF on glutamine synthetase (GS) and EAAC1 expression in the hippocampus of *Cdh5-Cre;Cdk5^f/f^* and control mice at 4 wk. **(E)** Quantitative analysis of protein levels for D was performed by densitometry (*n* = 5 mice per group; one-way ANOVA followed by Tukey’s multiple comparisons test). **(F)** Intrinsic membrane properties in the hippocampus of 4-wk-old *Cdh5-Cre;Cdk5^f/f^* and control mice treated with saline and CEF (*n* = 5 mice per group; one-way ANOVA followed by Tukey’s multiple comparisons test). **(G)** Microdialysis analysis for hippocampal glutamate concentrations in 4-wk-old *Cdh5-Cre;Cdk5^f/f^* and *Cdk5^f/f^* mice with saline and CEF treatment (*n* = 5 mice per group; ***, P < 0.001; one-way ANOVA followed by Tukey’s multiple comparisons test). **(H)** Simplified diagram of viral contracts for GLT1 overexpression in astrocytes and corresponding control virus. **(I and J)** Protein levels of GLT1-mCherry fusion were detected and quantified by Western blot (*n* = 3 mice per group; ***, P < 0.001; one-way ANOVA followed by Tukey’s multiple comparisons test). **(K)** Representative immunostaining images of mCherry (red) and astrocyte (green) illuminate the validity of GLT1 overexpression in astrocytes 3 wk after corresponding viral injection. Bar, 10 µm. **(L)** The seizure onset and duration were assessed in indicated groups (*Cdk5^f/f^* and *Cdh5-Cre;Cdk5^f/f^* mice separately stereotactically injected with indicated viruses: AAV-mCherry or AAV-GLT1 mixed with AAV-GFAP-Cre; *n* = 5 mice per group; *, P < 0.05; ***, P < 0.001; one-way ANOVA followed by Tukey’s multiple comparisons test). **(M)** Representative traces of sEPSC and mEPSC in indicated groups (*Cdk5^f/f^* + AAV-mCherry group, *Cdh5-Cre;Cdk5^f/f^* + AAV-mCherry group, *Cdk5^f/f^ +* AAV-GLT1 group, and *Cdh5-Cre;Cdk5^f/f^* + AAV-GLT1 group). **(N and O)** Cumulative distribution plots of the amplitudes and inter-event intervals of sEPSC (N) and mEPSC (O), with the insets depicting the average amplitudes and frequencies of sEPSC and mEPSC, in the hippocampus of four groups (*n* = 3–5 mice per group; ***, P < 0.001; one-way ANOVA followed by Tukey’s multiple comparisons test). **(P)** Representative GLT1 peak current incubated with DHK and quantification in indicated groups (*n* = 3–5 mice per group; *, P < 0.05; ***, P < 0.001; one-way ANOVA followed by Tukey’s multiple comparisons test). The numbers inside the bars represent the number of cells from three to five mice in each group. Error bars represent means ± SEM; n.s., not significant.

The above results demonstrated that the hyperexcitability of hippocampal neurons induced by endothelial *Cdk5* deficiency resulted from increased extracellular glutamate levels. Since astrocytes can take up glutamate and play an important role in glutamate homeostasis (Takahashi et al., 2015), we measured glial fibrillary acidic protein (GFAP) levels and found them to be significantly higher in the hippocampus but not in the cortex in endothelial *Cdk5* KO mice (Fig. 2, A and B). Immunostaining further showed a significant increase in the number of GFAP^+^ astrocytes in mutant mice compared with control mice (Fig. 2, C and D). Furthermore, the number of cells expressing both GFAP and S100β, an astrocytic marker associated with astrocyte proliferation and distress (Tynan et al., 2013), was also significantly increased in the hippocampus of 4-wk-old *Cdh5-Cre;Cdk5^f/f^* mice compared with *Cdk5^f/f^* mice (Fig. 2, E and F). However, no astrogliosis was observed in the cortex (Fig. S2 A). These results suggested that the hippocampus was more sensitive than the cortex to endothelial *Cdk5* deficiency.

Whole-cell recordings from hippocampal CA1 astrocytes showed significantly higher input resistance (R_in_: 20.29 ± 2.05 mΩ vs. 15.73 ± 1.03 mΩ), membrane time constants (τ_m_: 1.12 ± 0.21 ms vs. 0.70 ± 0.06 ms), and membrane capacitance (C_m_: 60.49 ± 8.59 pF vs. 43.26 ± 1.55 pF; Fig. 2 G), and no significant changes in resting membrane potential (RMP) in endothelial *Cdk5-*deficient astrocytes compared with control astrocytes of *Cdk5^f/f^* mice at 4 wk (−80.41 ± 0.71 mV vs. −80.38 ± 0.65 mV; Fig. 2 G). In *Cdh5-Cre;Cdk5^f/f^* mice, the increased C_m_ is consistent with the phenotype of astrogliosis as described previously (Hirrlinger et al., 2010; Iandiev et al., 2006).

To further determine whether endothelial *Cdk5* deficiency reduces astrocytic glutamate transport, N-methyl-D-aspartate, α-amino-3-hydroxy-5-methyl-4-isoxazolepropionic acid, and γ-aminobutyric acid type A receptors, as well as voltage-gated Na^+^ and Kir4.1 channels, were pharmacologically blocked (Fig. 2, H and I). Application of 100 µM glutamate induced an inward current, part of which was blocked by DL-threo-β-benzyloxyaspartic acid (TBOA), an inhibitor for glutamate/aspartate transporter (GLAST) and GLT1 glutamate transporters. In *Cdh5-Cre;Cdk5^f/f^* astrocytes, the TBOA-sensitive current was significantly reduced compared with *Cdk5^f/f^* astrocytes (122.20 ± 4.77 pA vs. 192.30 ± 7.30 pA; Fig. 2 H). Experiments with dihydrokainic acid (DHK), which blocked only GLT1 transporters, further showed that the difference in TBOA-sensitive current between the two groups was largely attributed to a decrease in the GLT1-mediated current (83.34 ± 1.34 pA [*Cdh5-Cre;Cdk5^f/f^*] vs. 148.70 ± 1.66 pA [*Cdk5^f/f^*]) but not the GLAST-mediated current (37.83 ± 4.77 pA [*Cdh5-Cre;Cdk5^f/f^*] vs. 43.60 ± 7.30 pA [*Cdk5^f/f^*]; Fig. 2 I). A decrease in GLT1-mediated current was also observed in *Cdh5-CreERT2;Cdk5^f/f^* mice (84.89 ± 4.88 pA [*Cdh5-CreERT2;Cdk5^f/f^*] vs. 153.40 ± 5.63 pA [*Cdk5^f/f^*]; Fig. S2 B) and BR1-iCre virus-injected *Cdk5^f/f^* mice (82.54 ± 3.06 pA [BR1-iCre-injected*-Cdk5^f/f^*] vs. 155.30 ± 4.63 pA [BR1-Con-injected-*Cdk5^f/f^*]; Fig. S2 C). Therefore, astrogliosis triggered by endothelial *Cdk5* deficiency weakened astrocytic GLT1-mediated current.

The β-lactam antibiotic ceftriaxone (CEF) is a potent GLT1 translational activator (Higashimori et al., 2016). Treatment with CEF increased GLT1 expression in the hippocampus 2.6-fold in *Cdh5-Cre;Cdk5^f/f^* mice and 2.3-fold in control mice. It had no effects on the expression of GLAST (Fig. 2, J and K), the astrocytic protein glutamine synthetase, or the neuronal glutamate transporter EAAC1 (Fig. S2, D and E). CEF delayed the onset (191.80 ± 34.02 s [CEF] vs. 89.33 ± 4.67 s [saline]) and shortened the duration (34.25 ± 10.86 s [CEF] vs. 106.00 ± 17.06 s [saline]) of PTZ-induced convulsions in 4-wk-old *Cdh5-Cre;Cdk5^f/f^* mice (Fig. 2 L). It also reversed the hyperexcitability of CA1 pyramidal neurons (Fig. 2, M and N) without affecting the intrinsic membrane properties of these neurons (Fig. S2 F). CEF increased the GLT1-mediated current in astrocytes, and the increase seen in *Cdh5-Cre;Cdk5^f/f^* mice (172.60 ± 1.99 pA vs. 87.06 ± 2.34 pA; Fig. 2, O and P) was significantly higher than that in *Cdk5^f/f^* mice (170.90 ± 2.14 pA vs. 156.40 ± 2.66 pA). CEF also decreased extracellular glutamate in both mutant and control mice (Fig. S2 G).

To further test whether restoration of GLT1 function reversed the effects of endothelial *Cdk5* deficiency, we injected an AAV expressing GFAP promoter-driven Cre-dependent GLT1-mCherry into the CA1 of *Cdk5^f/f^* and *Cdh5-Cre;Cdk5^f/f^* mice (Fig. S2, H–K). The treatment significantly delayed the onset of seizures (196.30 ± 19.10 s vs. 83.00 ± 16.26 s) and decreased seizure duration induced by PTZ (39.00 ± 6.75 s vs. 132.30 ± 13.91 s) in *Cdh5-Cre;Cdk5^f/f^* mice infected with AAV-GLT1 virus compared with AAV-mCherry infection (Fig. S2 L). AAV-GLT1 injection also reversed the increase in amplitudes and frequencies of sEPSC/mEPSC (Fig. S2, M–O) and the decrease in GLT1-mediated current (173.00 ± 5.58 pA vs. 86.49 ± 3.44 pA) in *Cdh5-Cre;Cdk5^f/f^* mice (Fig. S2 P).

Taken together, the above results suggested that the phenotypes observed in *Cdh5-Cre;Cdk5^f/f^* mice resulted, at least partially, from a decrease in GLT1 function in hippocampal astrocytes and that they can be reversed by increasing GLT1 expression.

### *Cdk5* deficiency induces overexpression of EC-derived CXCL1

**Figure 3.**
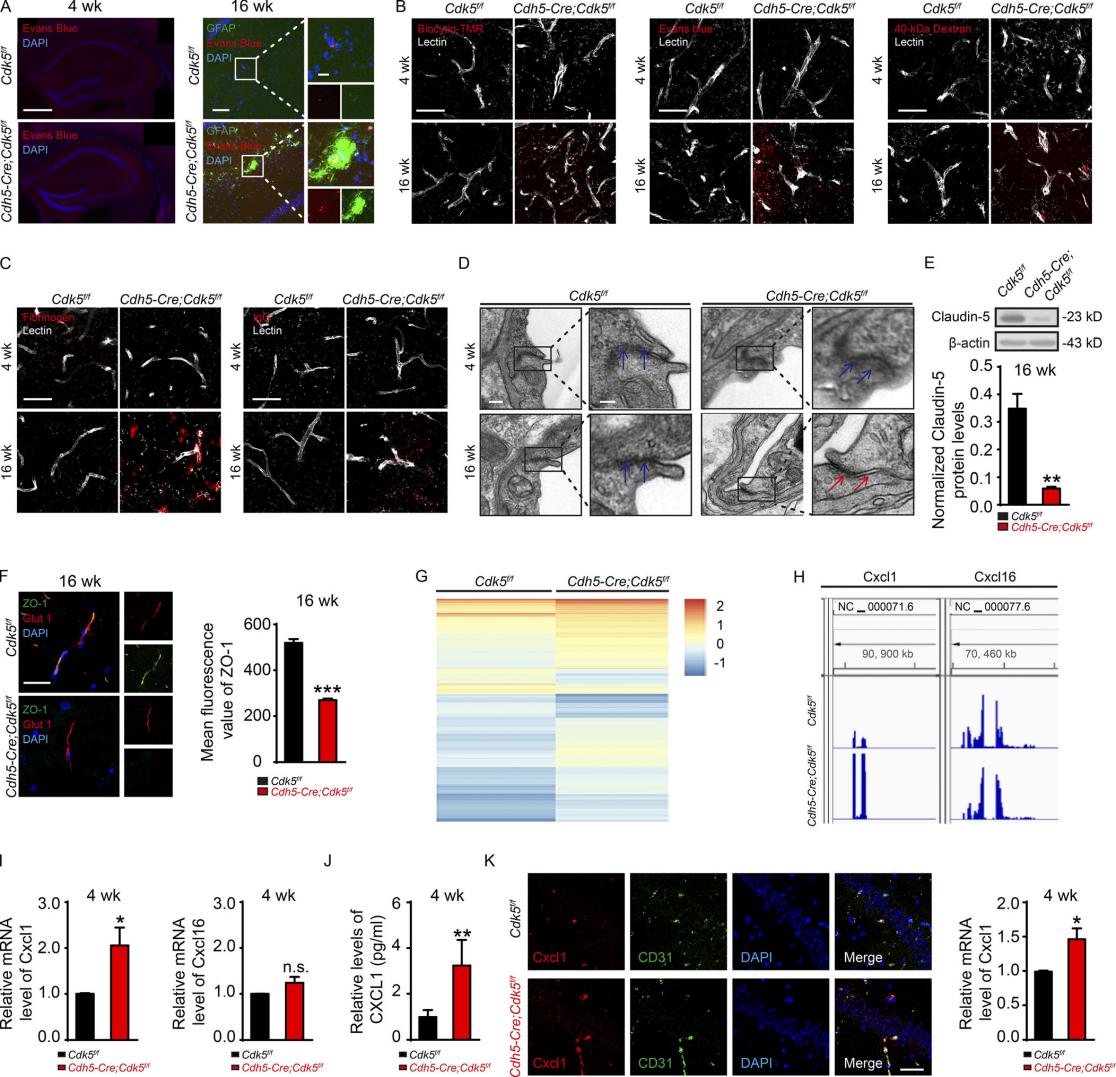
**The expression of the chemokine CXCL1 is increased in *Cdh5-Cre;Cdk5^f/f^* mice. (A)** Effect of endothelial *Cdk5* deletion on BBB leakage in mice at 4 and 16 wk. Left: Representative stitched images of immunostaining for Evans blue in the hippocampus in *Cdh5-Cre;Cdk5^f/f^* and *Cdk5^f/f^* mice at 4 wk (bars, 500 µm). The black represents areas that were not captured. Right: Representative images of immunostaining for GFAP (green) and Evans blue (red) in the hippocampus in *Cdh5-Cre;Cdk5^f/f^* and *Cdk5^f/f^* mice at 16 wk (bars, 100 µm; insets, 20 µm). DAPI staining is shown in blue. **(B)** Representative confocal microscopy images of Biocytin-TMR, Evans blue, 40-kD Dextran (red), and Lectin-positive microvessels (gray) in the hippocampus from *Cdh5-Cre;Cdk5^f/f^* and *Cdk5^f/f^* mice at 4 and 16 wk. Bars, 50 µm. **(C)** Representative confocal microscopy images of fibrinogen and IgG (red) and Lectin-positive microvessels (gray) in the hippocampus from *Cdh5-Cre;Cdk5^f/f^* and *Cdk5^f/f^* mice at 4 and 16 wk. Bars, 50 µm. **(D)** Transmission electron microscopy images of hippocampal endothelial tight junctions in samples from *Cdh5-Cre;Cdk5^f/f^* and *Cdk5^f/f^* mice at 4 and 16 wk. Blue arrows indicate tight junctions between ECs on vessels. Red arrows indicate gaps lacking tight junctions between ECs on vessels. Bars: main images, 200 nm; insets, 100 nm. **(E)** Representative immunoblots and quantification of the changes in tight junction proteins in the microvessels of *Cdh5-Cre;Cdk5^f/f^* and *Cdk5^f/f^* mice at 16 wk (*n* = 3 mice per group; **, P < 0.01; unpaired two-tailed Student’s *t* test). **(F)** Double immunostaining (left) for Glut1 (red) and ZO-1 (green) in the hippocampus from *Cdh5-Cre;Cdk5^f/f^* and control mice. Z-stacking through the microvessels was performed to confirm the colocalization of ZO-1 with endothelial marker Glut1. Bar, 25 µm. Quantification (right) of the ZO-1 fluorescence intensity in the vessels (*n* = 3 mice per group; ***, P < 0.001; unpaired two-tailed Student’s *t* test). **(G)** Heat map of all the differentially expressed genes in *Cdh5-Cre;Cdk5^f/f^* vs. control mice at 4 wk. The threshold was set to a fold change ≥ 2 and a *t* test P value ≤ 0.05. The data were standardized along the rows. **(H)** IGV genome browser view of the RNA sequencing profile from the analysis of 4-wk-old *Cdh5-Cre;Cdk5^f/f^* and *Cdk5^f/f^* mice. The Cxcl1 is shown up-regulated, and Cxcl16 is shown unchanged upon conditional KO Cdk5 in ECs. **(I)** The chemokine expression in primary cultured ECs from the cerebral microvessels of *Cdh5-Cre;Cdk5^f/f^* and *Cdk5^f/f^* mice at 4 wk was evaluated by qRT-PCR. Note that the Cxcl1 level was increased in *Cdh5-Cre;Cdk5^f/f^* mice, while that of another Cxcl family member, Cxcl16, was unchanged between *Cdh5-Cre;Cdk5^f/f^* and control mice. Cxcl1 and Cxcl16 mRNA levels were normalized to the corresponding Gapdh level (*n* = 5 mice per group; *, P < 0.05; unpaired two-tailed Student’s *t* test). **(J)** CXCL1 protein levels were detected through ELISA (*n* = 5 mice per group; **, P < 0.01; unpaired two-tailed Student’s *t* test). **(K)** RNAscope for Cxcl1 probes (red) and CD31 (an EC marker; green) in the hippocampal CA1 stratum pyramidale for the indicated groups. The DAPI counterstaining (blue) indicates the nuclei. The quantification of the relative Cxcl1 mRNA level is shown at the right (*n* = 3 mice per group; *, P < 0.05; unpaired two-tailed Student’s *t* test). Bar, 25 µm. The error bars represent means ± SEM. FC, fold change; n.s., not significant.

To investigate the role of the BBB in the above-described effects induced by endothelium-specific *Cdk5* KO, we analyzed BBB permeability and the expression of tight-junction proteins. BBB leakage, evidenced by Evans blue extravasation and positive immunostaining for astrogliosis, was present in 16-wk-old, but not 4-wk-old, *Cdh5-Cre;Cdk5^f/f^* mice (Fig. 3 A). Examination of exogenous tracer effusion (Fig. 3 B) and investigation of perivascular deposits of the plasma-derived proteins fibrinogen and IgG yielded similar results (Fig. 3 C). BBB disruption in 16-wk-old mutant mice was further confirmed by transmission electron microscopy (Fig. 3 D), immunoblotting for Claudin-5 (Fig. 3 E), and immunostaining for ZO-1 (Fig. 3 F). BBB damage is unlikely to be responsible for the decreased threshold for PTZ-induced seizures seen in 4-wk-old mice since it was not observed at that age.

**Figure S3.**
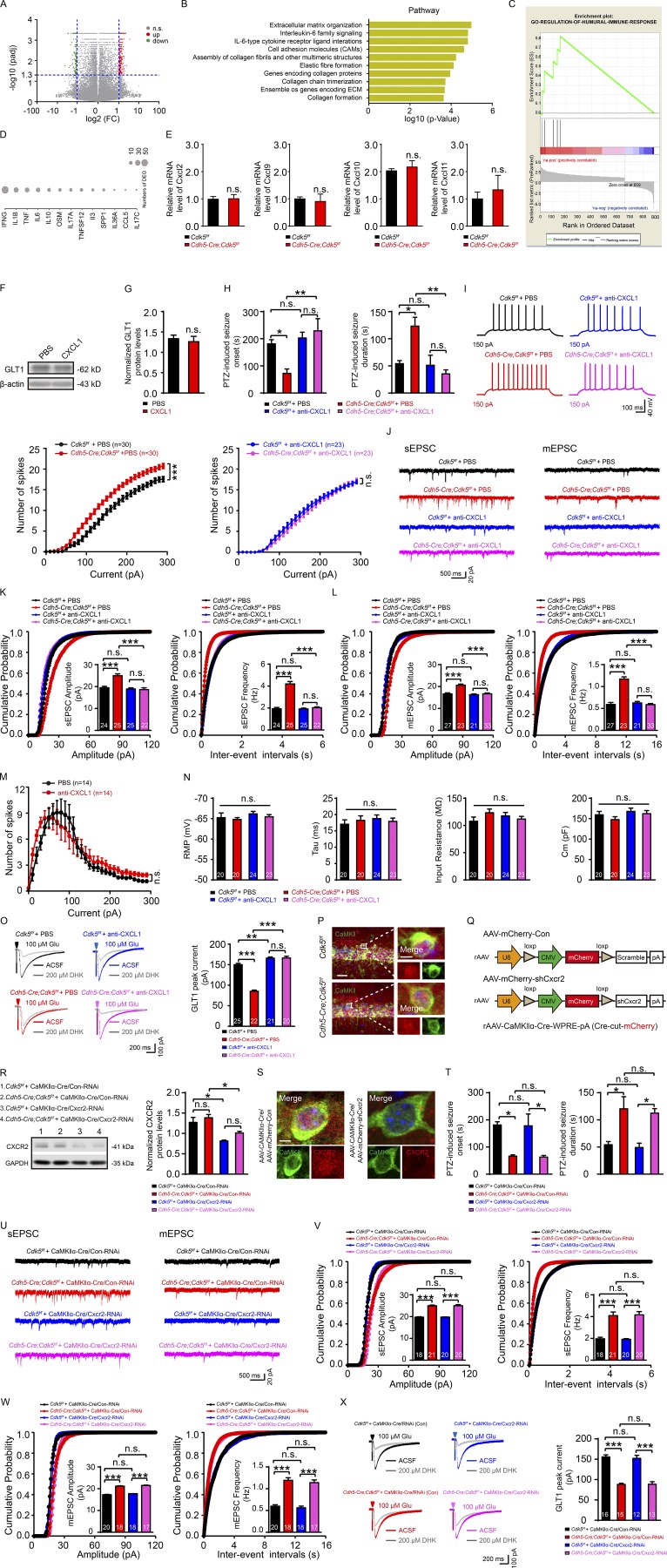
**Endothelial CXCL1 regulates astrocytic glutamate uptake via astroglial CXCR2 receptor. (A)** Volcano plots depicting fold change (FC) vs. P values for gene expression in *Cdh5-Cre;Cdk5^f/f^* compared with control mice at 4 wk. padj, P value that has been adjusted for the false discovery rate. **(B)** Relevant Kyoto Encyclopedia of Genes and Genomes pathways of differentially expressed genes in *Cdh5-Cre;Cdk5^f/f^* compared with control mice at 4 wk. ECM, extracellular matrix. **(C)** Enrichment plot for GO (Gene Ontology) regulation of the humoral immune response. **(D)** Most common upstream regulatory genes for the differentially expressed genes. **(E)** qRT-PCR of relative Cxcl2, Cxcl9, Cxcl10, and Cxcl11 mRNA levels in BMVECs of *Cdh5-Cre;Cdk5^f/f^* and *Cdk5^f/f^* mice at 4 wk. Cxcl2, Cxcl9, Cxcl10, and Cxcl11 mRNA levels were normalized to the corresponding *Gapdh* level (*n* = 5 mice per group; unpaired two-tailed Student’s *t* test). **(F and G)** Representative immunoblot (F) and quantification (G) demonstrated no changes on GLT1 protein levels in primary cultured astrocytes from 4-wk-old *Cdk5^f/f^* mice with CXCL1 incubation (*n* = 5 mice per group; unpaired two-tailed Student’s *t* test). **(H)** PTZ-induced seizure onset and duration were tested in *Cdh5-Cre;Cdk5^f/f^* and *Cdk5^f/f^* mice administered with CXCL1-neutralizing antibody (100 ng/ml) or not, with the PBS group as a control (*n* = 5 mice per group; *, P < 0.05; **, P < 0.01; two-way ANOVA followed by Tukey’s multiple comparisons test). **(I)** Representative APs and number of spikes in indicated groups: *Cdk5^f/f^* + PBS group, *Cdh5-Cre;Cdk5^f/f^ +* PBS group, *Cdk5^f/f^ +* anti-CXCL1 group, and *Cdh5-Cre; Cdk5^f/f^* + group (*n* = 5 mice per group; ***, P < 0.001; two-way ANOVA followed by Tukey’s multiple comparisons test). **(J)** Representative sEPSC (left) and mEPSC (right) recording traces in CA1 of hippocampus slices from *Cdh5-Cre;Cdk5^f/f^* and control mice treated with PBS or CXCL1-neutralizing antibody. **(K and L)** Cumulative distribution plots of the amplitudes and inter-event intervals of sEPSC (K) and mEPSC (L), with the insets depicting the average amplitudes and frequencies of sEPSC and mEPSC in indicated groups (*n* = 5 mice per group; ***, P < 0.001; unpaired two-tailed Student’s *t* test). **(M)** The number of APs by current injections from PBS- and CXCL1-antibody–treated primary cultured hippocampal neurons (*n* = 14 neurons; two-way ANOVA followed by Tukey’s multiple comparisons test). **(N)** RMP, τ_m_ (Tau), R_in_, and C_m_ in 4-wk-old *Cdh5-Cre;Cdk5^f/f^* and control mice treated with PBS and CXCL1-neutralizing antibody (*n* = 5–6 mice per group; unpaired two-tailed Student’s *t* test). **(O)** Representative GLT1 peak current incubated with DHK and quantification of GLT1 peak current in indicated groups (*n* = 5–6 mice per group; **, P < 0.01; ***, P < 0.001; one-way ANOVA followed by Tukey’s multiple comparisons test). **(P)** Representative immunostaining images of CXCR2 expression on pyramidal neurons in CA1 of *Cdh5-Cre;Cdk5^f/f^* and *Cdk5^f/f^* mice at 4 wk. Bars: main images, 100 µm; insets, 10 µm. **(Q)** Schematic of Cre-dependent AAV vectors for knockdown Cxcr2 on pyramidal neurons. **(R)** Representative Western blot of CXCR2 and quantification of CXCR2 expression in the hippocampus of *Cdh5-Cre;Cdk5^f/f^* and *Cdk5^f/f^* mice injected with the mixture of rAAV-CaMKIIα-Cre and rAAV-mCherry-Con or rAAV-mCherry-shCxcr2 (1:1 mixture) at 4 wk (*n* = 3 mice per group; *, P < 0.05; one-way ANOVA followed by Tukey’s multiple comparisons test). **(S)** Representative images of immunostaining on neuronal CXCR2 expression of hippocampus in *Cdk5^f/f^* mice respectively injected with the mixture of rAAV-CaMKIIα-Cre and rAAV-mCherry-Con or rAAV-mCherry-shCxcr2 (1:1 mixture). Bar, 5 µm. **(T)** The seizure onset and duration induced by PTZ were assessed in *Cdh5-Cre;Cdk5^f/f^* and *Cdk5^f/f^* mice both injected with the mixture of rAAV- CaMKIIα-Cre and rAAV-mCherry-Con or rAAV-mCherry-shCxcr2 (1:1 mixture; *n* = 5 mice per group; *, P < 0.05; one-way ANOVA followed by Tukey’s multiple comparisons test). **(U)** Representative sEPSC (left) and mEPSC (right) traces recorded in pyramidal neurons from hippocampal CA1 in indicated groups: *Cdk5^f/f^* + CaMKIIα-Cre/RNAi (Con) group, *Cdh5-Cre;Cdk5^f/f^* + CaMKIIα-Cre/RNAi (Con) group, *Cdk5^f/f^* + CaMKIIα-Cre/shCxcr2-RNAi group, and *Cdh5-Cre;Cdk5^f/f^* + CaMKIIα-Cre/shCxcr2-RNAi group. **(V and W)** Cumulative distribution plots of the amplitudes and inter-event intervals (right) of sEPSC (V) and mEPSC (W) from mice of the indicated groups, with the insets depicting the average amplitudes and frequencies of sEPSC and mEPSC (*n* = 3–5 mice per group; ***, P < 0.001; one-way ANOVA followed by Tukey’s multiple comparisons test). **(X)** Representative GLT1 transport current incubated with DHK in the hippocampus (left) and quantification of GLT1 transport current (right) in 7-wk-old mice in indicated groups (*n* = 5 mice per group; ***, P < 0.001; one-way ANOVA followed by Tukey’s multiple comparisons test). The numbers inside the bars represent the number of cells from three to six mice per group. Error bars represent means ± SEM; n.s., not significant.

Next, to identify the molecular mechanism that links endothelial *Cdk5* deficiency to astrogliosis, we cultured primary ECs from the cerebral microvessels of 4-wk-old *Cdh5-Cre;Cdk5^f/f^* and *Cdk5^f/f^* mice (Tan et al., 2019). Using gene expression microarrays combined with network analysis, we found that the transcripts that existed in primary ECs of both groups could be classified into two clusters (Fig. 3 G). Of the 20,210 robustly expressed genes, 986 expressed differentially between the mutant and control mice (Fig. S3 A). Most of these genes were up-regulated in the *Cdh5-Cre;Cdk5^f/f^* mice when compared with the *Cdk5^f/f^* mice. Expression analysis of Kyoto Encyclopedia of Genes and Genomes pathway analysis (Fig. S3 B) combined with Gene Ontology annotation (Fig. S3 C) and regulatory genes upstream of the differentially expressed genes (Fig. S3. D) showed that genes involved in IFNG, IL1B, TNF, and cell adhesion molecule events were related to endothelial activation (Berberich et al., 2011; Wu et al., 2015). Consistent with this activation pattern, several members of the Cxcl chemokine gene family were identified as differentially expressed, including Cxcl1, the level of which was found to be significantly increased in *Cdh5-Cre;Cdk5^f/f^* mice. No changes were found in the Cxcl16 level (Fig. 3 H).

Furthermore, these findings were confirmed by quantitative RT-PCR (qRT-PCR; Fig. 3 I) and ELISA assay (Fig. 3 J). The mRNA levels of Cxcl2, Cxcl9, Cxcl10, and Cxcl11 (Fig. S3 E) were unchanged in *Cdh5-Cre;Cdk5^f/f^* mice. In situ hybridization further showed that Cxcl1 mRNAs existed in ECs by using RNAscope (Fig. 3 K), suggesting its presence in ECs.

Overall, these data will be helpful for filling the current gaps in our knowledge, including those regarding the mechanisms of EC-induced aberrations in astrocytic function that eventually led to the hyperexcitability of pyramidal neurons that occurred during the pathological processes of spontaneous recurrent seizures.

### Aberrant elevation of EC-derived CXCL1 is the trigger of astrogliosis

**Figure 4.**
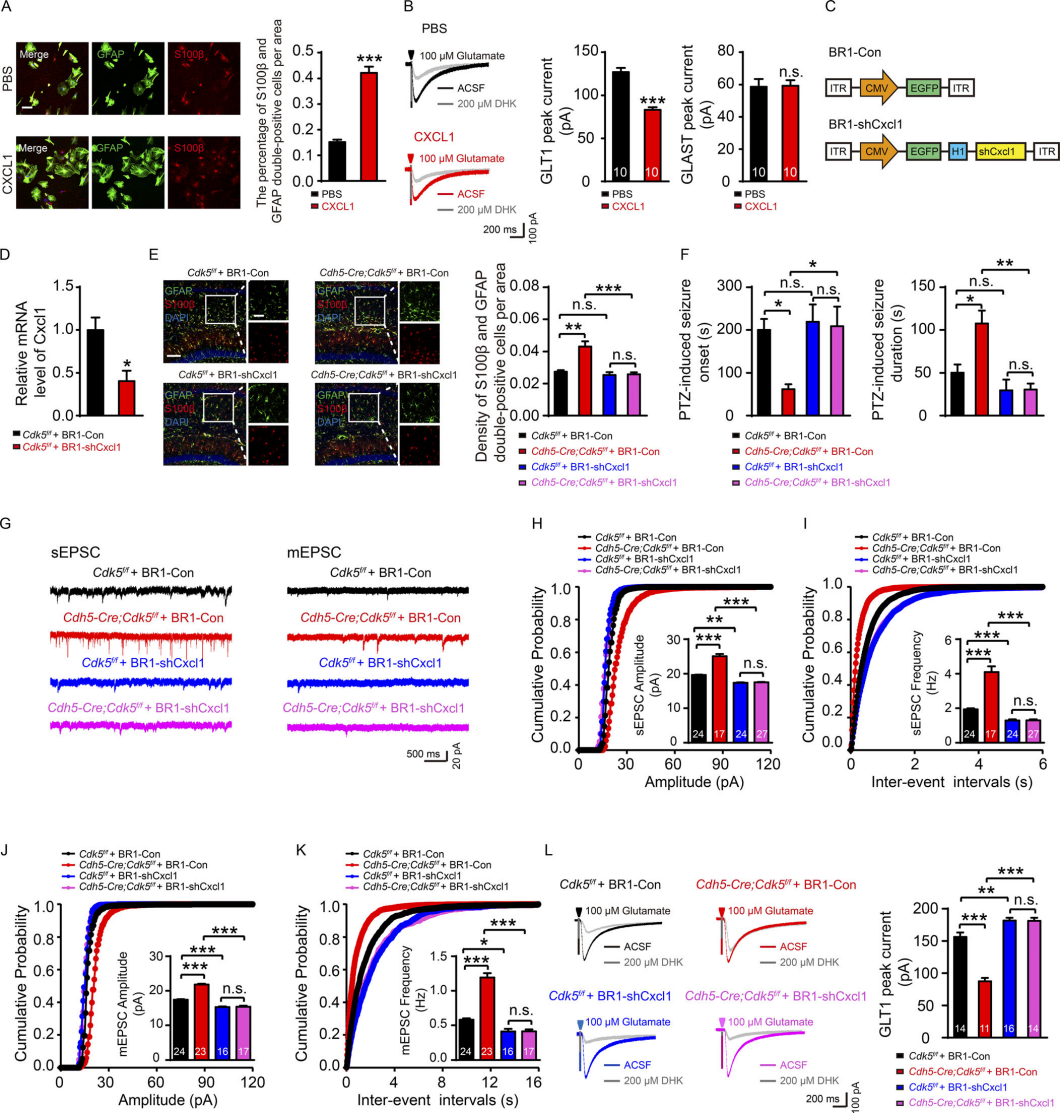
**Silencing of CXCL1 prevents the hyperexcitability of hippocampal neurons in endothelial *Cdk5-*deficient mice. (A)** Representative immunostaining images and quantification of GFAP (green) and S100β (red) in primary cultured hippocampal astrocytes from *Cdk5^f/f^* mice incubated with PBS and recombinant CXCL1 for 6 h (20 ng; *n* = 5 mice per group; ***, P < 0.001; unpaired two-tailed Student’s *t* test). Bar, 100 µm. **(B)** Glutamate uptake by primary cultured hippocampal astrocytes in the recombinant CXCL1 6-h incubation group and the control group was assessed after puff application of 100 µM glutamate for 500 ms before and after the application of 200 µM DHK (*n* = 5 mice per group; ***, P < 0.001; unpaired two-tailed Student’s *t* test). **(C)** Schematic representation of AAV-BR1 constructs indicating the inverted terminal repeats (ITR) at both ends and CMV promoter-driven EGFP (BR1-Con) or CMV promoter-driven shCxcl1 with EGFP (BR1-shCxcl1). **(D)** The relative mRNA level of Cxcl1 in primary cultured ECs from the cerebral microvessels was evaluated in BR1-Con– and BR1-shCxcl1–injected *Cdk5^f/f^* mice at 4 wk (*n* = 3 mice per group; *, P < 0.05; unpaired two-tailed Student’s *t* test). **(E)** Representative confocal images and quantification of GFAP (green) and S100β (red) in *Cdk5^f/f^* and *Cdh5-Cre;Cdk5^f/f^* mice injected with BR1-Con and BR1-shCxcl1 (*n* = 3 mice per group; **, P < 0.01; ***, P < 0.001; one-way ANOVA followed by Tukey’s multiple comparisons test). Bars: main images, 100 µm; insets, 50 µm. **(F)** PTZ-induced seizure onset latency and duration in *Cdk5^f/f^* and *Cdh5-cre;Cdk5^f/f^* mice injected with BR1-Con and BR1-shCxcl1 at 4 wk (*n* = 5 mice per group; *, P < 0.05; **, P < 0.01; one-way ANOVA followed by Tukey’s multiple comparisons test). **(G)** Traces showing sEPSC and mEPSC recorded in BR1-Con- and BR1-shCxcl1-injected mice at 4 wk. **(H–K)** Cumulative probability plots summarizing the mean sEPSC amplitudes (H) and sEPSC inter-event intervals (I) and cumulative probability plots summarizing the mean mEPSC amplitudes (J) and mEPSC inter-event intervals (K) in *Cdh5-Cre;Cdk5^f/f^* and *Cdk5^f/f^* mice injected with BR1-Con and BR1-shCxcl1 at 4 wk. The insets depict the average sEPSC and mEPSC amplitudes and frequencies (*n* = 3–5 mice per group; *, P < 0,05; **, P < 0.01; ***, P < 0.001; unpaired two-tailed Student’s *t* test). The data are presented as means ± SEM. **(L)** Representative GLT1 transport currents following DHK incubation in the hippocampus from 4-wk-old *Cdh5-Cre;Cdk5^f/f^* and *Cdk5^f/f^* mice with BR1-Con and BR1-shCxcl1 injection. Quantification of the GLT1 transport currents is shown on the right (*n* = 3–5 mice per group; **, P < 0.01; ***, P < 0.001; one-way ANOVA followed by Tukey’s multiple comparisons test). The numbers inside the bars represent the numbers of cells from three to five mice. The error bars represent means ± SEM; n.s., not significant.

To provide in vitro evidence showing that endothelial CXCL1 is necessary for the effects of *Cdk5* on astrocytes, we cultured hippocampal astrocytes from 3-wk-old *Cdk5^f/f^* mice. Application of recombinant CXCL1 protein (20 ng for 6 h) directly to cultured hippocampal astrocytes, which mimicked the effects of endothelial *Cdk5* KO, increased the number of cells expressing the astrocytic markers GFAP and S100β (Fig. 4 A), decreased the GLT1-mediated current (83.31 ± 3.00 pA vs. 126.90 ± 4.91 pA), and had no significant effects on the GLAST-mediated current (59.30 ± 3.51 pA vs. 58.75 ± 4.77 pA) or GLT1 expression (Fig. 4 B and Fig. S3, F and G).

To further test whether CXCL1 expression is a required step through which endothelial *Cdk5* KO induces astrogliosis and suppresses GLT1 function, we used shRNA to silence Cxcl1 expression in ECs in vivo (Fig. 4, C and D). Administration of AAV-BR1-shCxcl1, but not its control (AAV-BR1-Con), eradicated the astrogliosis in the *Cdh5-Cre;Cdk5^f/f^* mice (Fig. 4 E), delayed the onset (208.70 ± 46.20 s vs. 61.75 ± 11.80 s), and shortened the duration of PTZ-induced seizures (30.67 ± 6.89 s vs. 107.5± 15.18 s; Fig. 4 F). Cxcl1 silencing also reversed the increase caused by *Cdk5* deletion in the amplitudes and frequencies of sEPSC/mEPSC in CA1 pyramidal neurons (Fig. 4, G–K) and restored the GLT1-mediated current (*Cdk5^f/f^*: 181.80 ± 4.37 pA vs. 156.30 ± 6.81 pA; *Cdh5-Cre;Cdk5^f/f^*: 181.30 ± 4.71 pA vs. 87.48 ± 5.28 pA; Fig. 4 L).

We also studied the effect of a CXCL1-neutralizing antibody both in vivo and in vitro. The antibody delayed the onset (229.00 ± 44.77 s vs. 72.60 ± 16.02 s) and reduced the duration of PTZ-induced seizures (51.00 ± 18.50 s vs. 123.20 ± 16.44 s) in *Cdh5-Cre;Cdk5^f/f^* mice (Fig. S3 H). The treatment with the antibody also reversed the increase in excitability and in amplitudes and frequencies of sEPSC/mEPSC in CA1 pyramidal neurons (Fig. S3, I–L). It is unlikely that the CXCL1-neutralizing antibody acted directly on pyramidal neurons since the treatment had no effect on primary cultured hippocampal neurons (Fig. S3 M). It also had no effect on the intrinsic membrane properties of pyramidal neurons obtained from mutant or control mice (Fig. S3 N). CXCL1 neutralization also restored the GLT1-mediated current and eradicated the difference in *Cdh5-Cre;Cdk5^f/f^* mice (166.40 ± 4.22 pA vs. 85.32 ± 2.86 pA; Fig. S3 O).

These results provide further evidence for a role of endothelial CXCL1 in the phenotypes seen in endothelial *Cdk5* KO mice. These findings may provide insight into the interactions between ECs and astrocytes associated with synaptic homeostasis and/or pathological mechanisms of neurological disorders.

### Endothelial CXCL1 regulates astrocytic glutamate uptake via astroglial CXCR2 receptor

**Figure 5.**
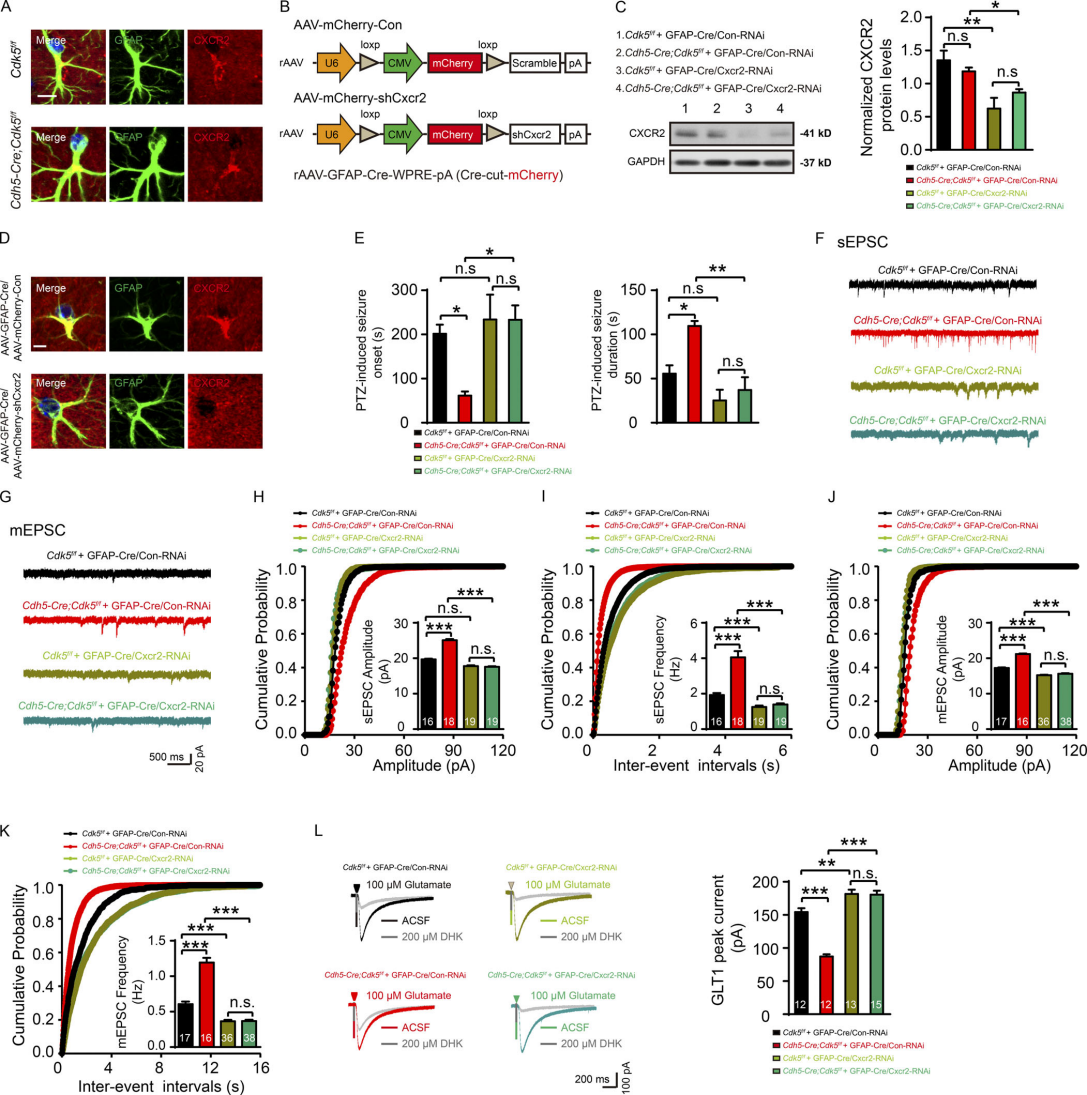
**Endothelial CXCL1 regulates astrogliosis and astrocytic glutamate uptake via CXCR2 in astrocytes. (A)** Representative immunostaining images of CXCR2 expression in astrocytes in the CA1 in *Cdh5-Cre;Cdk5^f/f^* and *Cdk5^f/f^* mice at 4 wk. Bar, 100 µm. **(B)** Schematic of GFAP-Cre-dependent AAV vectors for CXCR2 silencing. **(C)** Representative Western blot of CXCR2 and quantification of CXCR2 expression in the hippocampus from *Cdh5-Cre;Cdk5^f/f^* and *Cdk5^f/f^* mice injected with a virus mixture of rAAV-GFAP-Cre and rAAV-mCherry-Con or rAAV-mCherry-shCxcr2 (1:1 mixture) at 4 wk (*n* = 5 mice per group; *, P < 0.05; **, P < 0.01; one-way ANOVA followed by Tukey’s multiple comparisons test). **(D**) Representative images of immunostaining for astrocytic CXCR2 expression in the hippocampus from *Cdh5-Cre;Cdk5^f/f^* and *Cdk5^f/f^* mice injected with a virus mixture (1:1 mixture). Bar, 5 µm. **(E)** The onset latency and duration of seizures induced by PTZ were assessed in *Cdh5-Cre;Cdk5^f/f^* and *Cdk5^f/f^* mice after a virus mixture injection (*n* = 5 mice per group; *, P < 0.05; **, P < 0.01; one-way ANOVA followed by Tukey’s multiple comparisons test). **(F and G)** Representative sEPSC (F) and mEPSC (G) traces recorded in pyramidal neurons from the hippocampal CA1 regions of the indicated groups, including the *Cdk5^f/f^* + GFAP-Cre/RNAi (Con) group, the *Cdh5-Cre;Cdk5^f/f^* + GFAP-Cre/RNAi (Con) group, the *Cdk5^f/f^* + GFAP-cre/shCxcr2-RNAi group, and the *Cdh5-Cre;Cdk5^f/f^* + GFAP-cre/shCxcr2-RNAi group. **(H and I)** Cumulative probability plots of amplitudes (H) and inter-event intervals (I) of sEPSC from mice in the indicated groups. The insets depict the average sEPSC amplitudes and frequencies (*n* = 3–5 mice per group; ***, P < 0.001; one-way ANOVA followed by Tukey’s multiple comparisons test). **(J and K)** Cumulative probability plots of amplitudes (J) and inter-event intervals (K) of mEPC from mice in the indicated groups. The insets depict the average mEPSC amplitudes and frequencies (*n* = 3–5 mice per group; ***, P < 0.001; one-way ANOVA followed by Tukey’s multiple comparisons test). **(L)** Representative GLT1 transport currents following DHK incubation in the hippocampus from 4-wk-old mice in the indicated groups (left). Quantification of the GLT1 transport currents is shown on the right (*n* = 3–5 mice per group; **, P < 0.01; ***, P < 0.001; one-way ANOVA followed by Tukey’s multiple comparisons test). The numbers inside the bars represent the numbers of cells from three to five mice. The bars with error bars represent means ± SEM; n.s., not significant.

The biological effects of chemokines are mediated by G-protein-coupled chemokine receptors. CXCR2 is the primary receptor for CXCL1 (Brandenburg et al., 2016; Cao et al., 2016; Cao and Malon, 2018; Horuk et al., 1997; Liu et al., 2014). The interaction of CXCL1 with CXCR2 plays an important role in inflammation (Miyake et al., 2013). We confirmed its presence in both astrocytes and neurons (Fig. 5 A and Fig. S3 P). To determine which CXCR2 receptors are responsible for CXCL1’s effects, we used AAV2/9-GFAP-Cxcr2-RNAi and AAV2/9-CaMKIIα-Cxcr2-RNAi to silence Cxcr2 in astrocytes and pyramidal neurons, respectively (Fig. 5, B–D; and Fig. S3, Q–S).

Silencing of astrocytic Cxcr2 in *Cdh5-Cre;Cdk5^f/f^* mice resulted in longer latency (233.30 ± 32.66 s vs. 61.50 ± 9.19 s) and reduced the duration of PTZ-induced seizures (37.00 ± 14.62 s vs. 109.50 ± 5.81 s; Fig. 5 E). However, Cxcr2-specific silencing in pyramidal neurons had no effect on PTZ-induced seizures in *Cdh5-Cre;Cdk5^f/f^* mice (onset: 62.67 ± 6.06 s vs. 66.00 ± 5.13 s; duration: 112.30 ± 8.37 s vs. 120.30 ± 22.45 s; Fig. S3 T). Consistently, the differences in the amplitudes and frequencies of sEPSC and mEPSC between AAV-Con–injected *Cdk5^f/f^* mice and *Cdh5-Cre;Cdk5^f/f^* mice were eliminated in Cxcr2-RNAi–transducted astrocytes (Fig. 5, F–K), but not in Cxcr2-RNAi–transducted pyramidal neurons (Fig. S3, U–W). As shown in Fig. 5 L, Cxcr2-RNAi transduction restored the GLT1 current in the astrocytes (180.60 ± 5.97 pA vs. 87.24 ± 3.31 pA), whereas Cxcr2 knockdown in pyramidal neurons had no effect on GLT1 current in *Cdh5-Cre;Cdk5^f/f^* mice (88.49 ± 6.38 pA vs. 88.12 ± 2.97 pA; Fig. S3 X). These results support a working model in which CXCL1 reduces GLT1-mediated glutamate uptake by activation of CXCR2 receptors on astrocytes.

In summary, our findings reveal a previously unknown function of the endothelial-derived *Cdk5* signaling in the brain. Endothelial *Cdk5* deficiency induces spontaneous epilepsy in an age-dependent manner. The effect is associated with a decrease in GLT1-mediated glutamate uptake and an increase in excitability of hippocampal pyramidal neurons. Our evidence further suggests that these effects depend on CXCL1 release from ECs and subsequent activation of astrocytic CXCR2 receptors by CXCL1. Importantly, we found that these effects can be reversed by pharmacological restoration of GLT1 function (CEF), genetic silencing or immunoneutralization of CXCL1, or inhibition of the CXCL1 receptor CXCR2 on astrocytes. These findings warrant further investigation of endothelial *Cdk5* and its downstream pathways as potential new targets for the treatment of epilepsy.

## Materials and methods

### Mice and genotyping

Mice were housed under a 12-h light/dark cycle at a constant temperature of 22 ± 1°C with 40–60% humidity and provided access to standard food and water. The animals acclimated to their environment for ≥1 wk before the initiation of the experimental protocols. All experiments in animals were approved by the Committee for Animal Experiments at Nanjing Medical University and Zhejiang University in China.

Several mouse lines were used for our experiments. *Cdh5-Cre* mice (Cre expressed under the control of cadherin 5, also known as VEC-cre, stock no. 006137; The Jackson Laboratory) were crossed with mice carrying a loxP-flanked *Cdk5* gene (stock no. 014156; The Jackson Laboratory) to generate *Cdh5-Cre;Cdk5^f/f^* mice. Ai14 mice (Rosa26-tdTomato Cre reporter line, stock no. 007914; The Jackson Laboratory) and endothelial tamoxifen-inducible driver *Cdh5-CreERT2* mice (obtained from Prof. Ralf Adams, Max Planck Institute, Göttingen, Germany) were also used. To induce Cre activity at 4 wk, tamoxifen (10 mg/ml in ethanol/peanut oil; Sigma-Aldrich) was given in three consecutive i.p. injections (0.1 mg/g body weight) at postnatal day 28, 29, and 30. The numbers and ages of the mice used are indicated in the figure legends.

### Video/EEG recording of spontaneous seizures

Spontaneous seizure activity was monitored by EEG recording as described previously (Zhou et al., 2019). Mice were anesthetized and mounted in a stereotactic apparatus. For hippocampal recordings, bipolar twisted stainless steel electrodes (0.2 mm in diameter; Plastics One) were placed bilaterally in hippocampus CA1 (anteroposterior, −2.0 mm; mediolateral, ±1.5 mm; dorsoventral, −1.5 mm). Stainless steel screws (MX-0090-2; Plastics One) were placed epidurally and bilaterally over the frontal cortices, 0.5 mm posterior to the bregma and 2.45 mm lateral to the midline. An additional screw was placed just to the right of the frontal sinus and served as a reference electrode. The electrodes were then connected to a plastic pedestal (6 Channel; Plastics One), and the entire assembly was secured with dental cement. After 7 d of recovery from surgery, EEG recording was conducted continuously in freely moving mice with a Vanguard system (Lamont) at a sampling rate of 1 with a high-frequency filter of 70 Hz in synchronization with video recording for 24 h/d. Epileptic seizures were defined as field potentials twofold greater than the basal potential with durations longer than 10 s. Only mice with correctly located electrodes were included in the analysis.

### Gelatin-FITC imaging

Gelatin and FITC were diluted to a concentration of 1 mg/ml in sterile PBS as previously described (Underly et al., 2017). Mice were anesthetized and perfused with PBS and 4% paraformaldehyde (PFA; 40°C, 50 ml/animal) followed by gelatin-FITC (30°C, 30 ml/animal). After 2 h of incubation in ice, the heads were fixed in 4% PFA overnight. The next day, the brains were removed, fixed in 4% PFA for another 24 h, embedded in PBS, and cut into 35-µm sections on a vibratome. For immunofluorescent labeling, the sections were washed in PBS and incubated with DAPI for ∼20 min. After washing in PBS, the stained sections were examined with a confocal laser-scanning microscope as described previously (Jiang et al., 2017).

### PTZ-induced seizure test

4-wk-old mice were i.p. injected with PTZ in 0.9% saline at a dose of 60 mg/kg (injection volume 1 ml/100 g body weight) and individually placed in an acrylic box as previously described (Shen et al., 2016). Seizure behaviors were analyzed for 30 min after PTZ injection. The time to onset and the duration of tonic-clonic seizures were recorded. Behavior experiments were performed during the day between 09:00 and 12:00.

### Primary BMVECs

Primary BMVECs were isolated and cultured from 4-wk-old mice following a previous description (Rosas-Hernandez et al., 2018). Briefly, cortex tissue was dissected, then digested with gentle trituration every 10 min for 30 min at 37°C with DMEM containing 10 mM Hepes, 5 mM Ca^2+^, 10 mg/ml DNase I, and 400 U/ml collagenase. After adequate centrifugation at 1,000 *g* for 5 min, cell pellets were resuspended in 20% BSA and centrifuged at 1,000 *g* at 4°C for 20 min. Then, cell pellets were resuspended in EC culture medium (with puromycin) and seeded into plates coated with fibronectin. 2 d later, culture medium was altered with EC culture medium. For 10–12-d culture, cells were collected for further detections.

### BBB permeability assays

To assess BBB permeability, fluorescence tracers with different molecular size from small to large, including biocytin-tetramethylrhodamine (TMR; mol wt = 869 daltons; T12921; Thermo Fisher Scientific), Evans blue (mol wt = 960 daltons; E2129; Sigma-Aldrich), or TMR-dextran (mol wt = 40 kD; D1868; Invitrogen), were i.v. injected via the tail vein, respectively (Shi et al., 2016; Tan et al., 2019). 12 h later, the mice were anesthetized and transcardially perfused with 0.01 mol/liter PBS, followed by 4% PFA. The brains were collected and dehydrated in 30% sucrose in PBS, and frozen serial coronal brain sections (40 µm thick) were performed on a cryostat (Leica CM1900). Sections were processed for direct fluorescent detection of Alexa594. Images were acquired using a confocal fluorescence microscope (Zeiss LSM800). The regions of interest from the hippocampus were scanned at a resolution of 1,024 × 1,024 pixels with a 40× objective lens.

### Immunocytochemistry

Immunocytochemistry was performed as previously described (Wang et al., 2015). In brief, mice were anesthetized and transcardially perfusion fixed with 4% PFA in PBS. Horizontal brain slices (40 µm thick) were prepared by using a vibratome (Leica VT 1000 S), and sections were incubated for 30 min at room temperature with 0.1% Triton X-100 in PBS and for another 1 h in 3% BSA in PBS. For immunolabeling, the sections were incubated with indicated primary antibodies (anti-glucose transporter 1 [Glut1; 1:300, ab40084; Abcam], anti-S100β [1:500, ab52642; Abcam], anti-GFAP [1:500, MAB360; Millipore], anti-CDK5 [1:300, 2506; Cell Signaling Technology], anti-CaMKII [1:300, ab22609; Abcam], anti-ZO-1 [1:250, 402200; Invitrogen], anti-fibrinogen [1:200, ab34269; Abcam], anti-IgG [1:200, A-21203; Invitrogen], anti-Iba 1 [1:200, ab5076; Abcam], anti-NeuN [1:500, ABN78; Millipore], anti-Lectin [1:300, FL-1171; Vector], and anti-CXCR2 [1:200, ab14935; Abcam]) overnight at 4°C in the dark. Then, the sections were incubated with Alexa Fluor 488–conjugated anti-mouse IgG (1:300, A21202; Life Technologies), Alexa Fluor 594–conjugated anti-rabbit IgG (1:300, A21207; Life Technologies), and Alexa Fluor 488–conjugated anti-goat IgG (1:300, A11055; Life Technologies) for 1 h at 25°C. Images were acquired using a Zeiss LSM 800 confocal microscope.

### Western blot analysis

Western blot analysis was performed according to protocols as previously described (Lu et al., 2014). In brief, hippocampus tissues were separated and homogenized in lysis buffer. The equivalent amount of protein was subjected to SDS-PAGE gel (10–12%) and transferred to PVDF membranes (Millipore). The blots were probed with anti-GFAP (1:3,000, MAB360; Millipore), anti-GLAST (1:5,000, ab416; Abcam), anti-GLT1 (1:5,000, ab41621; Abcam), anti-EAAC1 (1:10,000, ab124802; Abcam), anti-glutamine synthetase (1:3,000, ab73593; Abcam), anti-Claudin-5 (1:2,000, 35–2500; Invitrogen), anti-GAPDH (1:5,000, 2118; Cell Signaling Technology,) and anti-β-actin (1:10,000, A1978; Sigma-Aldrich) at 4°C overnight, and then incubated with HRP-conjugated secondary antibodies. The proteins were visualized by an enhanced chemiluminescence detection system (Amersham Life Science). The density of protein bands was quantified using ImageJ software (US National Institutes of Health) and normalized to actin or GAPDH.

### Electrophysiology

Hippocampal and cortical slices were prepared with a vibratome (Leica VT 1000 S) in ice-cold cutting artificial cerebrospinal fluid (ACSF) containing (in mM) 125 NaCl, 3 KCl, 1.25 NaH_2_PO_4_, 2 MgSO_4_, 2 CaCl_2_, 25 NaHCO_3_, and 10 glucose, saturated with 95% O_2_ and 5% CO_2_, as previously described (Tan et al., 2019). After recovery for 30 min at 34°C in oxygenated ACSF, the slices were incubated at room temperature for ∼60 min. To measure the effect of the CXCL1-neutralizing antibody on electrophysiology recordings, the slices were incubated with 100 ng/ml CXCL1-neutralizing antibody (MAB4532; R&D Systems) in oxygenated ACSF for ∼2 h before the recording series.

Pyramidal neurons and astrocytes were visualized with an infrared-sensitive charge-coupled device camera with a 40× water-immersion lens (Olympus), and whole-cell patch-clamp recordings were performed (MultiClamp 700B Amplifier, Digidata 1440A analogue-to-digital converter). The patch-clamp intracellular solution used for pyramidal neurons contained (in mM) 130 potassium, 20 KCl, 10 Hepes, 4 Mg-ATP, 0.3 Na-GTP, 10 disodium phosphocreatine, and 0.2 EGTA (pH 7.25, adjusted with KOH; 288 mOsm). Tight seals were established using glass micropipettes with 3–8-MΩ open-pipette resistance at −70 mV for pyramidal neurons and at −80 mV for astrocytes. For current-clamp recording, action potentials (APs) were recorded under 750-ms suprathreshold current of 0–300 pA in 10-pA steps.

The passive membrane properties were recorded after obtaining the whole-cell configuration. R_in_ was induced with a rectangular hyperpolarizing current of −60 to 10 pA in 10-pA steps. τ_m_ was fit by an exponential function of the membrane potential change in response to rectangular hyperpolarizing current injection that induced small (3–5 mV) voltage deflections. C_m_ was obtained by dividing τ_m_ by *R*_in_.

To isolate sEPSC, 50 µM picrotoxin (PTX; Tocris Bioscience), a γ-aminobutyric acid type A receptor blocker, was added to the ACSF. mEPSCs were recorded in the presence of 50 µM PTX and 1 µM tetrodotoxin (TTX; Tocris Bioscience), which blocks sodium current. To isolate sIPSC, the bath solution containing 50 µM DL-AP5 (Tocris Bioscience; to block N-methyl-D-aspartate receptors) and 20 µM CNQX (Tocris Bioscience; to block α-amino-3-hydroxy-5-methyl-4-isoxazolepropionic acid receptors) was used. mIPSCs were recorded in the presence of 50 mM DL-AP5, 20 µM CNQX, and 1 µM TTX. The holding potential for EPSC recordings was −70 mV. Recordings were accepted under the condition that resistance was <20 MΩ.

Glutamate transport current was recorded according to the methods in previous reports (Armbruster et al., 2016; Armbruster et al., 2014; Diamond, 2005; Hanson et al., 2015). Briefly, for astrocytes, the recording pipettes were filled with a solution containing (in mM) 140 KCl, 0.5 CaCl_2_, 1 MgCl_2_, 5 EGTA, 10 Hepes, 3 Mg-ATP, and 0.3 Na-GTP (pH 7.2–7.3, adjusted with KOH; 288 mOsm). Astrocytes were identified by the following electrophysiological properties (Ge et al., 2006): an RMP of −80 mV and smaller R_in_, larger capacitance, and a more linear I-V curve than other types of glial cells. The identified astrocytes were used for glutamate uptake–mediated current analyses. In voltage-clamp mode, whole-cell patch-clamped astrocytes were held at −80 mV. To record the transport current, the following drugs were added to the bath solution: 50 µM DL-AP5, 20 µM CNQX, 50 µM PTX, 1 µM TTX, and 0.1 mM BaCl_2_ (Sigma-Aldrich). Then, 100 µM glutamate was applied by puff application for 500 ms to activate transport current. TBOA (0.3 mM; Tocris Bioscience) was used to isolate the total (GLT1+GLAST) glutamate transport current, and 100 µM DHK (Tocris Bioscience) was used to isolate the GLT1-mediated current. All analyzed and presented transport currents are TBOA or DHK subtractions. Data acquisition and analysis were performed with pClamp 10.3 software.

### In vivo microdialysis experiments

A unilateral guide cannula was implanted into hippocampal CA1 regions (anteroposterior, −2.0 mm; mediolateral, ±1.5 mm; dorsoventral, −1.5 mm) of *Cdk5^f/f^* and *Cdh5-Cre;Cdk5^f/f^* mice as described previously (Berger et al., 2018). After 7 d of recovery, a microdialysis probe (AZ-2-01) was inserted and secured to the guide cannula for dialysate collection. After 90 min of ACSF circulation at a rate of 1 µl/min, dialysis samples were gathered every 20 min for three times at a rate of 0.5 µl/min with a microinfusion pump (ESP-32), and the glutamate concentrations were analyzed using an HPLC system.

### CEF and vehicle treatment

Mice were i.p. injected with a 200-mg/kg body weight dose of CEF (Rocephin; Roche) dissolved in 0.9% saline from 09:00 to 10:00 for 5 consecutive d as described previously (Hefendehl et al., 2016). Control groups of *Cdh5-Cre;Cdk5^f/f^* and *Cdk5^f/f^* mice were given equivalent injections of 0.9% saline for 5 consecutive d.

### Drug microinjection by guide cannula

Bilateral hippocampus cannulas were implanted in targeted coordinates of hippocampus CA1 (anteroposterior: −2.0 mm; mediolateral: ±1.5 mm; dorsoventral: −1.5 mm) in 4-wk-old mice (John et al., 2012). After 7-d recovery from surgery, a CXCL1 neutralizing antibody (1 µg/2 µl) was administered via the guide cannula using a micro syringe pump at a rate of 46 nl/s according to instructions.

### Transmission electron microscopy

Transmission electron microscopy was performed as previously described (Wang et al., 2017a). Mice were anesthetized and transcardially perfused with 4% PFA in PBS. The brain was removed and fixed with 4% PFA at 4°C overnight. Horizontal brain slices (200 µm thick) were prepared using a vibratome (Leica VT 1000 S). The target hippocampal tissues were cut into 0.5 × 0.5-cm squares, fixed with 2.5% glutaraldehyde at room temperature, and incubated to 4°C overnight. The sections were postfixed in 1% osmium tetroxide for 1 h; gradient-dehydrated in 50%, 70%, 90%, and 100% ethanol; and embedded in epoxy resin. Polymerization was confirmed before the blocks were cut into ultrathin sections with an ultramicrotome. The sections were viewed under an electron microscope (Hitachi 7000; Nikon) after being stained with uranyl acetate and lead citrate.

### High-throughput mRNA sequencing

Primary cultured EC samples from *Cdk5^f/f^* and *Cdh5-Cre;Cdk5^f/f^* mice were collected for total RNA extracts using TRIzol Reagent (Invitrogen) followed by quality check on an Agilent 2100 Bioanalyzer (Agilent Technologies), a NanoDrop spectrophotometer (Thermo Fisher Scientific), and a 1% agarose gel. Qualified samples, which had an RNA integrity number >7, were retained for next-generation library preparation according to the manufacturer’s protocol (an NEBNext Ultra RNA Library Prep Kit for Illumina). RT and cDNA modification were performed as described previously (Best et al., 2019). After that, DNA sequencing of the libraries was performed on an Illumina sequencer, and data were analyzed by GENEWIZ.

### RNA isolation and real-time qPCR

Mouse brain ECs were isolated as described above (Navone et al., 2013). The samples were homogenized, and total RNA was isolated using RNAiso Plus (Takara). cDNA was synthesized using a PrimeScript RT Reagent Kit with gDNA Eraser (Takara) in accordance with the manufacturer’s instructions. qRT-PCR was performed with EvaGreen 2× qPCR MasterMix-No Dye (abm) to detect the mRNA expression level of Cdk5, Cxcl1, Cxcl2, Cxcl9, Cxcl10, Cxcl11, Cxcl16, and Gapdh (as an internal control). The details of primer sequences are as follows: Cdk5 (forward), 5′-CAA​TGC​AGA​AAT​ACG​AGA​AAC​TGG-3′; Cdk5 (reverse), 5′-CTT​TGA​GTA​GAC​AGA​TCT​CCC​G-3′; Cxcl1 (forward), 5′-ACC​GAA​GTC​ATA​GCC​ACA​CTC-3′; Cxcl1 (reverse), 5′-CTC​CGT​TAC​TTG​GGG​ACA​CC-3′; Cxcl2 (forward), 5′-GGC​CAC​CAA​CCA​CCA​GGC​TA-3′; Cxcl2 (reverse), 5′-TTC​CGT​TGA​GGG​ACA​GCA​GCC-3′; Cxcl9 (forward), 5′-GCT​CTG​CCA​TGA​AGT​CCG​CTG​T-3′; Cxcl9 (reverse), 5′-GCA​ATT​GGG​GCT​TGG​GGC​AA-3′; Cxcl10 (forward), 5′-AGC​GCT​TCA​TCC​ACC​GCT​GA-3′; Cxcl10 (reverse), 5′-GGG​CAG​GAT​AGG​CTC​GCA​GG-3′; Cxcl11 (forward), 5′-CCC​GAG​TAA​CGG​CTG​CGA​CA-3′; Cxcl11 (reverse), 5′-GGG​CTC​ACA​GTC​AGA​CGT​TCC​C-3′; Cxcl16 (forward), 5′-TGG​CAC​CCA​GAT​ACC​GCA​GG-3′; Cxcl16 (reverse), 5′-ATG​TGC​AGG​GGT​GCT​CGT​GT-3′.

### CXCL1 ELISA

The CXCL1 protein levels of primary cultured ECs extracted from *Cdk5^f/f^* and *Cdh5-Cre;Cdk5^f/f^* mice were detected with mouse CXCL1/KC Quantikine ELISA Kit (R&D Systems; Wang et al., 2017b; Taki et al., 2018). In brief, samples were incubated for 2 h and washed with Quantikine wash buffer. The absorbance was measured at 450 nm with a 540-nm correction, and the concentrations were calculated according to the manufacturer’s protocols.

### RNAscope in situ hybridization assay

RNA in situ hybridization assay was performed to detect the expression of endothelial Cxcl1 mRNA transcripts using an RNAscope Multiplex Fluorescent Reagent Kit v2 (Advanced Cell Diagnostics; Pacher et al., 2018; Valenta et al., 2018) according to the manufacturer’s recommendations with RNAscope Probe-Mm-Cxcl1 (407721; Advanced Cell Diagnostics) and RNAscope Probe-Mm-Pecam1 for ECs (316721; Advanced Cell Diagnostics). The signal was developed by tyramide signal amplification reagents (PerkinElmer). The fluorescent signals were captured with a confocal fluorescence microscope (Zeiss LSM800).

### Primary astrocyte culture

All procedures for primary astrocyte cultures were performed as previously described (Sun et al., 2017). Briefly, hippocampal tissues from 4-wk-old mice were digested in 0.25% trypsin at 37°C for 20 min. Tissue homogenate was centrifuged with a 23% percoll solution at 4°C for 15 min, and the precipitate was retained. The precipitate was resuspended and preincubated with DMEM and 10% FBS for 15 min; then, cells were seeded onto plates coated with poly-D-lysine. Half of the medium was replaced every 3 d. After 10 d, culture plates were shaken continuously for 24 h at 37°C to eliminate microglia. The cells were split into new plates at a density of 30,000 cells/cm^2^ and incubated for subsequent experiment.

### Virus injection

Viral injections were performed in *Cdk5^f/f^* and *Cdh5-Cre;Cdk5^f/f^* mice. rAAV-CaMKIIα-Cre-WPRE-pA (2.50 × 10^12^ viral particles ml^−1^) or rAAV-GFAP-Cre-WPRE-pA (5.54 × 10^12^ viral particles ml^−1^) mixed with rAAV-U6-Loxp-CMV-mCherry-Loxp-shRNA (Cxcr2, 1:1; 2.20 × 10^12^ viral particles ml^−1^) was injected bilaterally into CA1 (anteroposterior: −2.0 mm; mediolateral: ±1.5 mm; dorsoventral: −1.5 mm) with corresponding control virus. The sequence of Cxcr2 shRNA in AAV-Cxcr2-RNAi is 5′-CGA​AAT​CCT​GTT​AAG​GTA​AAC​CTT-3′. Similarly, rAAV-Efla-DIO-GLT1-mCherry-WPRE-pA (5.43 × 10^12^ viral particles ml^−1^) was injected bilaterally into CA1 of 4-wk-old mice. All viruses were purchased from BrainVTA. Viruses were injected in a volume of 400 nl at 100 nl/min as previously described (Tan et al., 2019). To minimize tissue injury, the AAVs were delivered into the target region last for 15 min through a 10–20-μm diameter tip of a glass microelectrode with a nanoliter injector (WPI).

For systematic delivery of AAV-BR1 (plasmid provided by Jakob Körbelin, University Medical Center Hamburg-Eppendorf, Hamburg, Germany), 4-wk-old mice were i.v. injected in the lateral tail vein with AAV-BR1-CAG-iCre-2A-EGFP or AAV-BR1-CAG-EGFP or pAKD-CMV-bGlobin-EGFP-H1-shRNA (Cxcl1; 1.6 × 10^11^ genomic particles/mouse). The sequence of Cxcl1 shRNA in BR1-Cxcl1-RNAi is 5′-CCA​CTG​CAC​CCA​AAC​GAA​GTC​ATA-3′. Further detections were performed 3 wk after virus injection.

### Statistical analysis

Data are presented as means ± SEM. Unpaired two-tailed Student’s *t* test was used for datasets including two independent groups. One-way ANOVA, followed by Tukey’s post hoc test, was applied to analyze different groups when there were more than two. Two-way ANOVA (genotype × trial) was used to analyze groups with two factors. P < 0.05 was considered to be statistically significant.

### Data availability

Sequence data that support the findings of this study have been deposited in the National Center for Biotechnology Information Sequence Read Archive with the accession codes SRR9637648, SRR9637649, SRR9637650, SRR9637651, SRR9637652, and SRR9637653.

### Online supplemental material

Fig. S1 shows that ablation of endothelial *Cdk5* increased sensitivity to PTZ-induced epilepsy. Fig. S2 shows that dysfunction of astroglial GLT1 contributes to neuronal excitability and epilepsy. Fig. S3 shows that endothelial CXCL1 regulates astrocytic glutamate uptake via astroglial CXCR2 receptor. Video 1 shows an episode of a spontaneous behavioral seizure observed in 16-wk-old *Cdh5-Cre;Cdk5^f/f^* mice.
